# A wide-ranging review of galvanic vestibular stimulation: from its genesis to basic science and clinical applications

**DOI:** 10.1007/s00221-025-07079-8

**Published:** 2025-04-27

**Authors:** Sarah Marchand, Alba Langlade, Quentin Legois, Alexandra Séverac Cauquil

**Affiliations:** 1 Centre de Recherche Cerveau et Cognition, Université de Toulouse, CNRS, Toulouse, France; 2 Service d’ORL, Otoneurologie et ORL Pédiatrique-CHU Toulouse Purpan, Toulouse, France

**Keywords:** Galvanic vestibular stimulation, Posture, Eye movements, Neuro-imagery, Percept, Clinical

## Abstract

Galvanic vestibular stimulation (GVS) involves applying small electrical currents to the vestibular organs via electrodes placed on the mastoids, providing a powerful tool for investigating vestibular function. Despite its long history, GVS remains highly relevant for researchers due to its ability to probe the vestibular system’s role in posture, gaze control, perception, and cortical processing. Recent technical advances have considerably expanded its application in both basic research and clinical practice. Despite the fact it is not realistic to cover all aspects of GVS within the constraints of a manuscript, this narrative review summarizes the history and neurophysiological mechanisms of GVS and provides new insights and perspectives for current and future studies, both in fundamental and clinical applications. We synthesize the main findings from neurophysiological, behavioral, and neuroimaging studies, focusing on the effects of GVS on postural control, ocular responses, cortical activity, and self-motion perception. Then diagnostic and therapeutic applications are explored in balance disorders, stroke rehabilitation, and neurodegenerative diseases. Clinical approaches could benefit from greater reliance on laboratory research to refine stimulation protocols, for maximum efficacy in its therapeutic use. A final discussion summarizes what is currently well-established with regard to GVS and opens up new and exciting perspectives in basic science and clinical applications.

## Introduction

Galvanic vestibular stimulation (GVS) has provided unique insights into vestibular function for over a century. The use of electrical current to explore the physiology of biological structures is not a new approach. The earliest descriptions of using electrical currents for therapeutic purposes can be traced back to ancient Greece, where Aristotle and Plato documented the ability of the torpedo fish, a species capable of naturally generating electrical currents, to produce curative effects. This method was later used during the Roman Empire to help a patient suffering from a headache (Althaus 1873; Sarmiento et al. 2016).

Centuries later, the scientific application of electrical currents took a significant leap forward with the work of Galvani (1791). He demonstrated that stimulating nerves and muscles with galvanic currents could evoke muscle contractions, laying the foundations for understanding electrical excitation in biological tissues. Building on this breakthrough, von Humboldt adapted the method for use in humans. von Humboldt (1797), he demonstrated the functional connections between nerves and muscles. These early advancements quickly led the scientific community to recognize the value of electrical currents as an essential tool for exploring neuronal functions, still widely used in neuroscience today.

In 1800, Volta further explored galvanic stimulation by applying his newly invented battery to his ears. He reported sensations of spinning, imbalance, and a boiling sound, suggesting that galvanic currents could influence sensory systems beyond nerves and muscles, including the vestibular and auditory systems (Volta 1923). Purkinje (1819), expanded on these findings by observing balance disturbances and equilibrium disruptions when passing currents through the head. Initially attributed to general effects of electrical stimulation, these phenomena were later linked specifically to the vestibular system.

Decades later, Hitzig documented nystagmus as a response to applying an electric current to the brains of dogs and humans. His observations provided the first clear evidence that galvanic stimulation could modulate vestibular motor outputs (Hitzig 1874). In the same year, Breuer advanced this research by combining galvanic stimulation with labyrinthectomy in animals, confirming that responses such as head movements and balance disruptions originated from the vestibular system (Breuer 1875). Both Hitzig and Breuer experimented with galvanic stimulation on themselves, reporting sensations similar to those described by Volta. However, they provided more detailed descriptions, noting a distinct sensation of falling toward the side of the cathode (Camis 1930).

Nowadays, the use of GVS in humans has not fundamentally changed; the principle remains the same. Electrodes are placed on the mastoid processes rather than directly in the ears, usually with the anode positioned on one mastoid process and the cathode on the other. This bipolar configuration, often referred to as bilateral stimulation, allows the electrical current to flow through the skull, engaging both vestibular organs then the surrounding neural structures (see Sect. "Functional anatomy of the vestibular system". on the functional anatomy of the vestibular system) before reaching the opposite electrode (Ertl and Boegle 2019). GVS has been widely used to modulate vestibular activity in a controlled manner without requiring head movement, significantly contributing to our current understanding of the vestibular system and its functions. Research using GVS has explored ocular responses (Watson et al. 1998a; MacDougall et al. 2002, 2003; Séverac Cauquil et al. 2003; Jahn et al. 2003b; Aw et al. 2006), postural adjustments (e.g. Nashner and Wolfson 1974; Lund and Broberg 1983; Fitzpatrick and Day 2004), and cortical correlates of motion and self-motion perception (e.g. Lopez et al. 2012), in both healthy subjects and patients. These studies have paved the way for clinical applications and demonstrated the potential of GVS in therapeutic settings. Numerous reviews have already highlighted the popularity and utility of GVS in both research and clinical contexts (e.g. Fitzpatrick and Day 2004; Curthoys 2010; St George and Fitzpatrick 2011; Dlugaiczyk et al. 2019; Pires et al. 2022; McLaren et al. 2022, 2023), mainly covering the somatic projections of the vestibular system, even though the autonomous part of the vestibular system was also addressed (Yates et al. 2014). The most recent ones focus rather on specific aspects of GVS such as noisy GVS (nGVS, e.g. McLaren et al. 2022, 2023), parameter settings (Valter et al. 2025), posture and gait (Xie et al. 2024) or rehabilitation (e.g. Pires et al. 2022). Alternatively, the present review aims at providing an overview of GVS (1) principles, (2) experimental data across various domains (i.e. posture, gaze, neuroimaging, perception) and (3) clinical applications in a broad domain related to spatial orientation. One of the objectives is to bridge the gap between research in basic science and clinical applications. This led us to preferentially select studies on somatic aspects, pertaining to the body position and/or movement, using directional square and/or pulse GVS, largely explored in basic science. Noisy GVS, that now predominates in clinical settings, have been reported in a dedicated section.

## The vestibular system and its activation by galvanic stimulation

### Functional anatomy of the vestibular system

Since this review focuses on somatic aspects of the effect of GVS on the vestibular system, we will, in this section, only cover relevant anatomical elements within these limits. For an in-depth analysis of the links between the vestibule and vegetative functions, we refer to articles exploring vestibulo-sympathetic responses (Yates et al. 2014) or the role of the insular cortex in autonomic regulation (Nagai et al. 2021).

The vestibular apparatus is a peripheral sensory organ, which comprises the posterior membranous labyrinth, and central pathways that include both peripheral and central components (Sakka and Vitte 2004). The peripheral vestibular system is composed of two functional entities: the three semicircular canals, and the otolith organs (Iurato 1967; Kingma and van de Berg 2016). The semicircular canals are arranged orthogonally to detect head rotation in all three planes, while the otolith organs, utricle and saccule, are sensitive to translational movements and static head position relative to gravity. Together, these systems form a robust framework for detecting gravitational forces as well as angular and linear accelerations, which are vital for maintaining equilibrium and spatial navigation.

The vestibular nerve, containing two types of vestibular afferents: regular and irregular afferents (Goldberg and Fernandez 1971; Eatock et al. 2008; Eatock and Songer 2011) enters the brainstem at the pontomedullary junction, where it projects to the vestibular nuclei (Ramon y Cajal 1985; Büttner-Ennevera 1999; Conrad et al. 2014). The vestibular nuclei are central integration hubs where proprioceptive, visual, cerebellar, striatal and reticular afferents converge, ensuring that vestibular information is integrated with other sensory modalities as soon as it reaches the central nervous system (Sakka and Vitte 2004; Angelaki and Cullen 2008).

Vestibular nuclei give rise to ascending fibers directed towards the cerebellum, reticular formation, thalamus, and cortex, as well as descending fibers going to the spinal cord. Vestibular information reaches the thalamus through ipsilateral and contralateral brainstem pathways: the contralateral pathways integrate ocular motor control, and ipsilateral pathways primarily convey perceptual information (Conrad et al. 2014). The vestibular thalamus comprises several thalamic nuclei (Liedgren et al. 1976; Magnin and Fuchs 1977; de Waele et al. 2001; Meng et al. 2007; Marlinski and McCrea 2008), and receives input from the vestibular nuclei and the medial pulvinar (Shinder and Taube 2010). Most neurons of the vestibular thalamus encode muscular proprioceptive signals but not visual or oculomotor information (Deecke et al. 1974; Magnin and Fuchs 1977; Marlinski and McCrea 2008; Shinder and Taube 2010).

Ascending projections from the vestibular nuclei to the thalamus are organized into two pathways: one involves the anterior thalamic nuclei, which provide cues about head direction and spatial orientation in the hippocampus and to retrosplenial and entorhinal cortices (Shinder and Taube 2010), the second is the posterior pathway, which originates in the ventral posterior lateral (VPL) thalamus, receiving a multimodal input, including vestibular, somatosensory, proprioceptive, visual and motor signals (Marlinski and McCrea 2008; Meng and Angelaki 2010; Cullen 2019), conveying self-motion information to the cortex for motor planning and sensory integration (Nagata 1986; Matesz et al. 2002).

The literature on the anatomy of the vestibular system, particularly regarding its somatic dimension, is extensive and provides details about its peripheral and central components. However, it remains largely descriptive and does not necessarily address how these anatomical elements interact functionally under specific conditions, such as during the application of GVS. It is therefore essential to explore the neural substrate of GVS, and particularly where and how electrical stimulation activates the vestibular system.

### Neural substrate of GVS: where and how electrical current activates the vestibular system?

Because GVS is extensively used in both basic and applied scientific paradigms, it is crucial to understand and master how it interacts with the nervous system. This relies on animal studies, here are reported the most significant for achieving an acceptable comprehension of GVS mode of action.

#### Direct afferent versus hair cell stimulation

Early hallmark animal studies on squirrel monkeys by Goldberg et al. (1982, 1984) suggested that GVS directly activates vestibular nerve afferent fibers at the spike trigger zone, bypassing hair cell synapses. However, later studies comparing nerve discharge induced by GVS in the presence or gentamicin-induced absence of hair cells, indicated that hair cells might also be recruited (Norris et al. 1998; de Waele et al. 2002; Cheng et al. 2010). However, the use of gentamicin must be interpreted with caution as it affects not only vestibular hair cells but also neuronal fibers. Indeed, additional evidence supporting the activation of vestibular hair cells by GVS was provided by an in vitro pharmacological study on tadpoles by Gensberger et al. (2016). This study showed that GVS recruits both vestibular nerve afferents (currents from 0.15 mA) and hair cells at the lowest current intensities.

#### Otolithic organs versus semi-circular canals stimulation

Some studies have suggested a dominant contribution of the otolithic organs to ocular responses elicited by GVS, with minimal involvement of the semicircular canals (Cohen et al. 2012), others have argued the opposite, proposing a greater role for the semicircular canals and limited involvement of the otolithic organs (Fitzpatrick and Day 2004). Today, the scientific consensus indicates that both structures contribute to vestibular responses during GVS. This conclusion was confirmed by recent insights provided by Cullens’ group. Indeed, Kwan et al. (2019), and more recently Forbes et al. (2023) directly investigated single vestibular afferent responses to GVS applied to the mastoid processes, exactly as is done in humans, of awake-behaving monkeys. Doing so, they ended a long-lasting controversy about which part of the vestibular apparatus was stimulated by GVS from interpretation of either its behavioural (e.g. Zink et al. 1998; Séverac Cauquil et al. 2003; Fitzpatrick and Day 2004) or electrophysiological consequences in animal studies where the current is delivered directly in the inner ear (e.g. Goldberg et al. 1982; Ezure et al. 1983; Courjon et al. 1987; Kim and Curthoys 2004). Using a setup similar to the one used on humans, these authors demonstrated that transmastoid GVS produces robust and parallel activation of both regular and irregular afferents, with similar thresholds for canal and otolith afferents (Kwan et al. 2019).

#### Asymmetries of fiber modulation

Cathodal and anodal galvanic currents onsets were found to increase and decrease afferent fibers firing respectively, when applied within the inner ear in squirrel monkey (Goldberg et al. 1982) or guinea pig (Kim and Curthoys 2004) and when applied externally in macaques (Kwan et al. 2019; Forbes et al. 2023). All studies showed that cathodal and anodal galvanic currents offsets trigger opposite firing modulation: a decrease or increase afferent fibers firing, respectively. Cathodal current steps trigger action potentials in afferent fibers and anodal currents silence vestibular afferents (Goldberg et al. 1984). Importantly, Forbes et al. (2023) confirmed on externally stimulated macaques the asymmetry previously demonstrated in guinea pigs stimulated with the ear: excitation obtained from cathodal onset or anodal offset is larger than the depression from cathodal offset or anodal onset (Kim and Curthoys 2004).

This asymmetry is larger for irregular than for regular fibers, and even though both types respond to GVS, the irregular, thicker fibers are more sensitive and responsive to the current: the correlation between GVS intensity and the activated fiber type was described in several studies on various animal models (Kim and Curthoys 2004; Kim et al. 2011; Gensberger et al. 2016; Forbes et al. 2023).

#### Stimulus shape implications

When current steps are applied, an immediate transient increase in discharge is followed by a sustained and variably elevated firing rate that depends on the afferent fiber type (Kim and Curthoys 2004; Forbes et al. 2023). Sinusoidal currents are often described as corresponding more closely to the velocity than the position component of head movements with similar phase relations between GVS-and motion-evoked responses in VOR neuronal elements (Gensberger et al. 2016), therefore more suitable to stimulate a natural head motion. Gain and phase increase for frequencies above 1 Hz; regardless the recorded afferent fiber type, with gain being stronger for irregular fibers and cathodal currents (Forbes et al. 2023). These authors observed similar irregular versus regular and cathodal versus anodal asymmetries for sinusoidal and stochastic currents than for constant currents.

In noisy or stochastic currents, subthreshold band-limited noisy current are thought to add stochastic resonance to the peripheral vestibular system, caused by resonance of hair cell and afferent responses (Indresano et al. 2003; Flores et al. 2016) increasing the spontaneous activity of irregular afferents and therefore improve the performance of the vestibular system in peripheral sensory processing. As for DC currents, Forbes et al. (2023) found vestibular afferent response nonlinearities to stochastic GVS.

### The different GVS configurations

Valter et al. (2025) very recently reviewed the GVS parameter settings in clinical applications, in order to clarify the choice of the appropriate configuration depending on the clinical purpose. This valuable way of thinking can be extended to basic science paradigms.

**Fig. 1:**
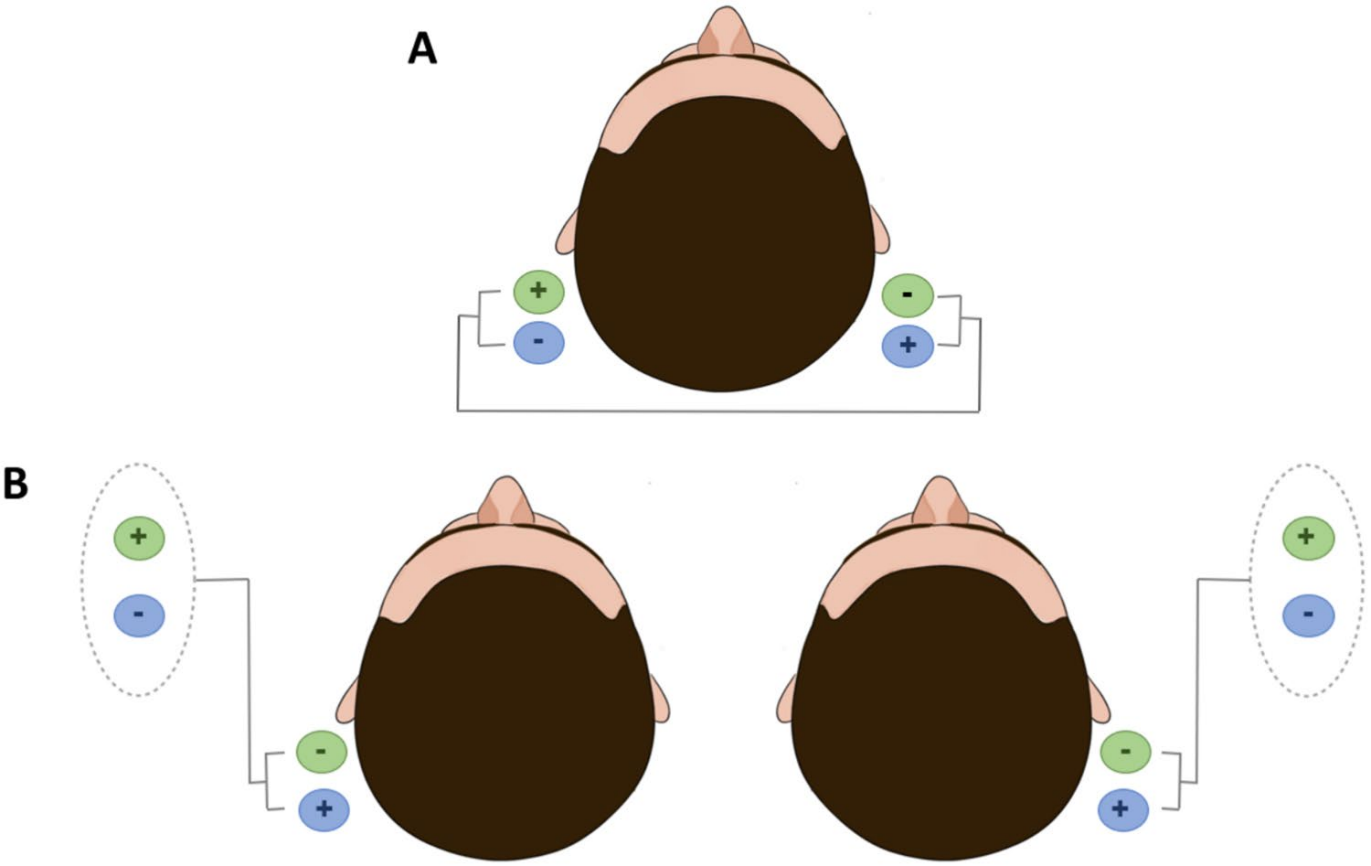
One-path montages. **A** Binaural bipolar montage: bilateral bipolar stimulation: anode placed on one mastoid and the cathode on the other side. **B** Monaural montage: cathode or anode on the mastoid and the electrode of opposite polarity on the seventh cervical vertebra, the forehead, vertex, or forearm

From the first GVS studies, the binaural bipolar setup, with anodes and cathodes placed over the two mastoid processes, respectively (Fig. 1A), has been extensively used, giving rise to a body tilt or eye mouvement in the anode direction (e.g. Hitzig 1874; Babinski 1901; Dzendolet 1963). A monaural alternative setup was used early on: positive or negative stimulation was applied over one mastoid process, with a second electrode completing the circuit at another part of the head or body. Examples include placements on the seventh cervical vertebra (Rosengren et al. 2009; Park et al. 2015; Xavier et al. 2023), the forehead (Séverac Cauquil et al. 1998, 2000; Murofushi et al. 2002), the vertex (Watson and Colebatch 1998), or the ipsilateral forearm (Coats 1972; Smetanin et al. 1990) (Fig. 1B). The details of postural and oculomotor outcomes of these studies, mainly deviations and sway towards the anode, are discussed in Sect. "Cortical correlates of GVS" and "Perceptual effects of GVS", respectively.

**Fig. 2:**
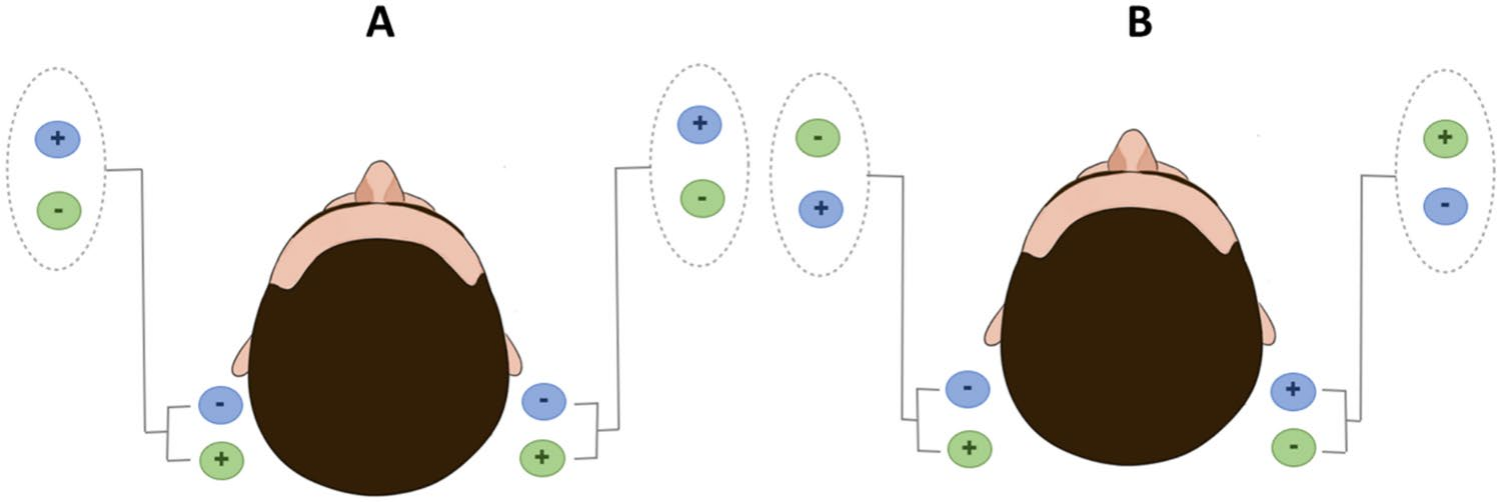
Two-path montages. **A** Binaural monopolar montage: right and left mastoid receive the same polarization, either anodal (green) or cathodal (blue patches) while the completing electrode is placed either on forehead, wrist or neck. **B** Opposite double monaural monopolar montage: right and left mastoid receive opposite polarization, either anode left-cathode-right (green) or cathode-left anode-right (blue patches) while the completing electrode is placed on the forehead

Also, some studies have described the use of a binaural monopolar setup (Fig. 2A) to successfully and consistently orientate the GVS response (and then input?) along the anteroposterior axis (Njiokiktjien and Folkerts 1971; Krizkova and Hlavačka 1994; Séverac Cauquil et al. 1998, 2000; Aoyama et al. 2015; Aedo-Jury et al. 2020). In this configuration, the right and left mastoids receive the same polarization, either anodal or cathodal, while the completing electrode is placed on the forehead (Séverac Cauquil et al. 1998, 2000; Aoyama et al. 2015; Aedo-Jury et al. 2020) or the wrist (Krizkova and Hlavačka 1994). The other double monaural GVS, adding opposite polarization of the mastoids (Fig. 2B), was so far even less used than the former (Séverac Cauquil et al. 1998, 2000; Aoyama et al 2015), yet still providing responses superimposable to binaural bipolar ones, in both postural and cerebral activation studies (Séverac Cauquil et al. 2000; Aedo-Jury et al. 2020).

This section demonstrates that GVS constitutes both a research topic in its own right as well as a research tool and a lot has been learned about the mode of action of this old tool, until very recently. Based on the studies reported in this section, it is now established that cathodic currents increase afferent firing of all branches of the vestibular nerve, hence all part of the vestibular apparatus, while anodic decrease it, in an asymmetric fashion that favors excitation and irregular fibers. Such non-linearities must be taken into account in future studies, in particular the ones investigating directional responses. In that respect, the orientation of the GVS-induced vestibular input, and as a consequence the different responses (ocular, whole-body or cerebral) must be expected as a result of the discrepancy in polarization on one or both sides.

## GVS in basic science

### GVS as a tool to study posture and gait

Postural responses to GVS have been extensively studied over the years. The primary postural response is a sustained body sway directed toward the anode (Coats 1972). At the start of stimulation, the body leans toward the anodal side, and within 1–2 s, this movement stabilizes, leaving the body in a tilted position. Once the stimulation ceases, all body segments gradually return to their original alignment (Coats 1972; Britton et al. 1993; Day et al. 1997; Séverac Cauquil et al. 2000; Cathers et al. 2005). Consistent responses are observed during walking, with deviations toward the anodal side when subjects walk with closed eyes (Fitzpatrick et al. 1999; Bent et al. 2000; Abbariki 2024). Several researchers have sought to explain the postural response induced by GVS (Fitzpatrick et al. 1994; Hlavacka et al. 1995; Day et al. 1997; Wardman et al. 2003a, b).

**Fig. 3:**
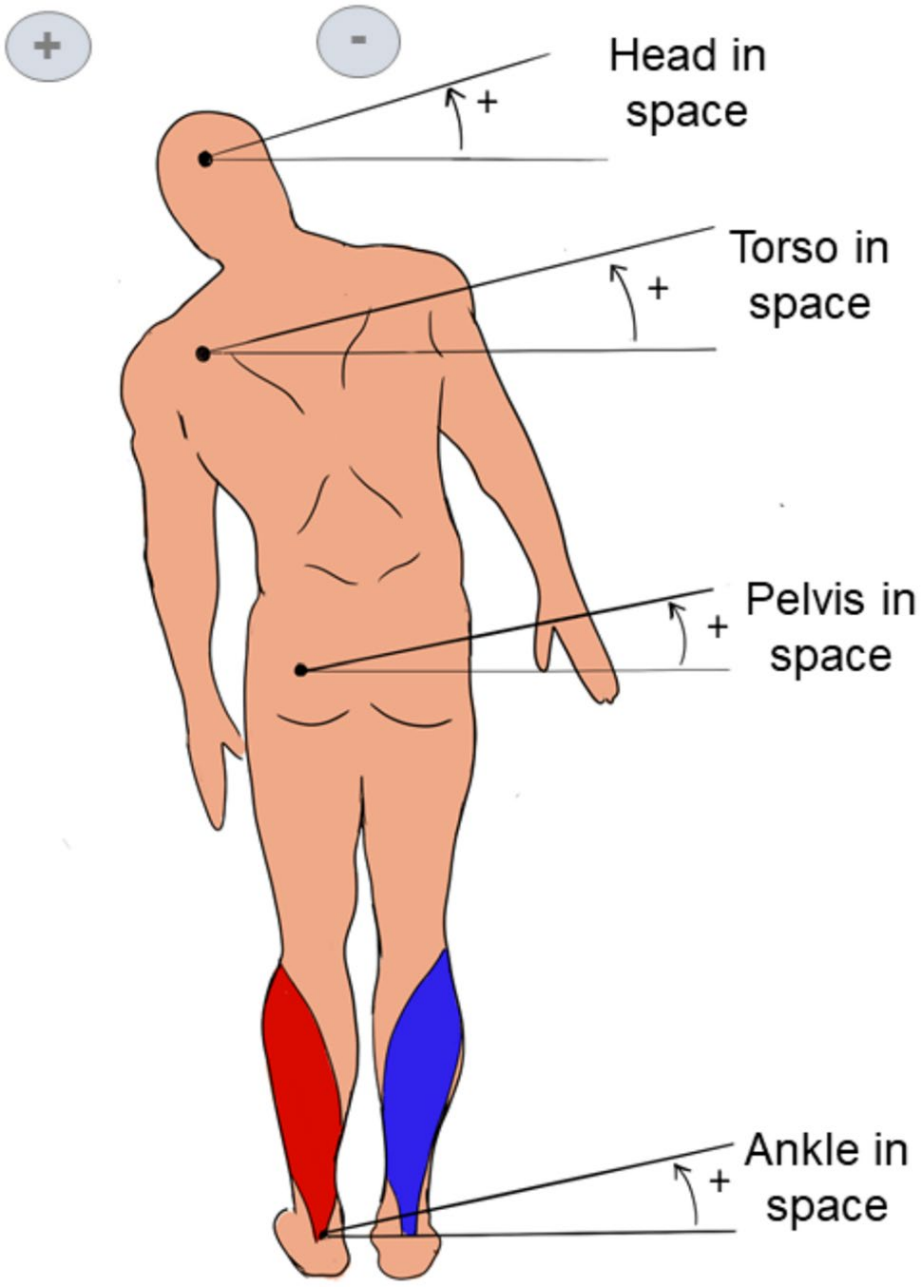
Typical postural response with sway of the ankles, pelvis, trunk, and head under bipolar binaural GVS (cathode on the right mastoid, anode on the left mastoid), inspired by Day et al. (1997), while the subject looks forward. This response is associated with soleus muscle activity based on Tokita et al. (1989), showing increased activation on the left soleus (red) and decreased activation on the right soleus (blue)

One prominent hypothesis, proposed by Fitzpatrick et al. (1994), suggests that the postural response arises from a compensatory effect triggered by an illusion of movement in the opposite direction (toward the cathode). However, studies investigating muscle responses timing have challenged this idea (Britton et al. 1993; Day et al. 1997). These studies highlight two distinct components of the postural response, each with opposite polarities, in response to GVS (Britton et al. 1993). The short-latency component, characterized by its small amplitude and brief duration, occurs approximately 50–60 ms after stimulation onset. Interestingly, its polarity opposes the direction of body sway and is only measured at higher stimulation intensities (Day et al. 1997). In contrast, the medium-latency component has a longer duration, with a latency of around 120 ms, and aligns with the direction of body sway. By contrasting head orientation with respect to gravity, Cathers et al. (2005) showed that short-latency components arise from the otolithic organs while inputs from the SCCs induce the medium-latency response. More recent evidence from Phillips et al. (2013) further supports the canal specific contributions to postural responses. Using localized electrical stimulation of individual semicircular canals, they demonstrated that each canal produces distinct sway directions. These canal-specific responses confirm the differential contributions of otoliths and canal inputs initially highlighted by Cathers et al. 2005. Taken together, these findings support the notion that the sway response to GVS comprises distinct functional components, each associated with specific vestibular inputs. The sway response is indeed actually divided into two different components: a step-like response, hence driven primarily by otolith afferents, and a ramp-like response, driven by canal afferents (Wardman et al. 2003a). The presence of these two components points to the involvement of the vestibulospinal pathway (Baldissera et al. 1990), which operates independently of perceptual processes. Additionally, the difference in their latencies indicates the involvement of distinct central pathways, such as the vestibulospinal and reticulospinal tracts, thought to mediate these responses (Britton et al. 1993; Cathers et al. 2005). Another explanation for the postural response is that its primary function is to maintain the body vertical by minimizing postural muscle activity. This mechanism relies on the integration of vestibular, proprioceptive, and visual inputs to generate a unified estimate of body orientation. GVS disrupts vestibular input, triggering a compensatory realignment of the body to maintain balance (Inglis et al. 1995; Hlavacka et al. 1995). Day et al. (1997) provided a detailed description of these coordinated postural adjustements, involving all body segments: the head tilts relative to the trunk, the trunk shifts over the pelvis, and the pelvis adjusts relative to the ground (Fig. 3). Interestingly, the tilt of the legs is typically about half the magnitude of the tilt observed in the upper body (Wardman et al. 2003b). Day et al. (1997) further proposed that GVS might be interpreted by the body as a tilt of the support surface. This idea is supported by the observation that movements similar to those elicited by GVS naturally occur when the support surface tilts (Purdon Martin 1967). However, findings from Wardman et al. (2003b) suggest differing responses: GVS elicits greater bending when standing on foam, while platform tilt results in more pronounced bending when feet are apart, accompanied by opposite lower body movement (Wardman et al. 2003b). These discrepancies may be explained by rapid sensory reweighting dynamics, as proposed by Assländer and Peterka (2016). Indeed, sensory contributions are not fixed, but rather rapidly rebalanced following sudden changes in available sensory information, affecting postural amplitude, direction, and timing.

#### Factors modulating the postural response

Head orientation is a key determinant of sway direction: turning the head to the left shifts the response from a lateral plane to an anteroposterior plane, indicating a head-centered reference frame of the body response (Nashner and Wolfson 1974; Lund and Broberg 1983; Hlavacka and Njiokiktjien 1985; Britton et al. 1993; Pastor et al. 1993; Fitzpatrick and Day 2004).

Balance-engagement of the muscle is essential for generating postural responses. EMG recordings reveal that responses in lower body muscles disappear when the subject is seated but upper body muscles reponse persist if these are involved in maintaining balance (Britton et al. 1993). While seated, the postural response remains present in the trunk and head, though it is less pronounced compared to standing, and the response in the legs vanishes entirely (Day et al. 1997).

Sensory interactions, involving visual and somatosensory inputs, also play a significant role in modulating the postural response to GVS. Deprivation of visual or somatosensory inputs amplifies postural response amplitude (Coats 1972; Britton et al. 1993; Fitzpatrick et al. 1994; Horak and Hlavacka 2001; Day et al. 2010). For instance, postural and muscular responses are amplified when eyes closed and feet positioned close together (Day et al. 1997), and diminished when feet are put apart (Day et al. 1993, 1997). Sensory interactions also influence the shape of response observed: ramp-like responses when there is no sensory interaction, plateau-like responses when visual or somatosensory inputs conflict with vestibular input (Cathers et al. 2005). These conflicting signals modulate the contribution of vestibular input, thereby interrupting the continuous response (Wardman et al. 2003a; Cathers et al. 2005). This type of plateau response has been observed in a subject with impaired sensory afferents, such as in cases of deafferentation, but with significantly greater amplitudes (Day and Cole 2002). Sensory interactions during GVS have also been studied in walking subjects (Fitzpatrick et al. 1999; Bent et al. 2000; Abbariki 2024). Deviation toward the anode is absent with open eyes, highlighting the stabilizing effect of visual inputs (Fitzpatrick et al. 1999). Additionally, slower walking speeds result in greater deviations, reflecting an increased reliance on vestibular input, whereas faster walking relies more on preplanned motor programs, reducing the influence of vestibular and proprioceptive inputs (Fitzpatrick et al. 1999; Bent et al. 2000).

Current intensity modulates both postural and muscular responses to GVS: response amplitudes increase with the intensity (Coats 1972; Petersen et al. 1994; Fitzpatrick et al. 1994), with the largest increase occurring between the torso and pelvis (Day et al. 1997). In walking subjects, deviations toward the anode also increase proportionally with current intensity (Bent et al. 2000). Some studies have identified a linear relationship between current intensity and the displacement amplitude of the center of pressure (Popov et al. 2005). However, this relationship is better described by a power law (exponent 0.55), suggesting a nonlinear transfer between sensory input and motor output (Day et al. 2010). For instance, doubling the stimulus current results in only 73% of the expected response increase. This nonlinearity may provide a functional benefit by amplifying small signals while preventing saturation during larger inputs (Day et al. 2010). These findings appear to align with Forbes et al. (2023), discussed in Sect. «The different GVS configurations » of this review, who reported greater sensitivity and stronger activation of irregular vestibular afferents with increasing current intensity. Although Forbes et al. (2023) did not explicitly quantify a non-linear power-law relationship, their results highlight differential fiber responses consistent with a nonlinear transfer function between current amplitude and neural activation.

Movements induced by brief pulses are restricted to the head relative to the trunk, suggesting the involvement of the medial vestibulospinal tract rather than the lateral one (Fitzpatrick and Day 2004). This goes along with the fact that high frequency movements rarely occur for natural whole-body movement, but rather for head-on-trunk motion. Brief pulses of 2 ms elicit biphasic EMG responses in the ipsilateral sternocleidomastoid and bilaterally in the masseter muscles (Watson and Colebatch 1998; Deriu et al. 2003). These responses primarily involve irregular vestibular afferents, which have lower activation thresholds (Smith and Goldberg 1986), see part 1-anatomy. Sinusoidal stimulation produces frequency-dependent sway, with higher frequencies (> 1 Hz) leading to increased instability, particularly in the lateral direction (Coats 1972; Hlavacka and Njiokiktjien 1985; Petersen et al. 1994). At last, stochastic GVS, has been shown to induce mediolateral sway, primarily within the 1–2 Hz range, while minimizing anteroposterior responses, highlighting the frequency-dependent nature of postural control (Pavlik et al. 1999), but also the directionality of the montage (see part 1). Pink noise enhances vestibular sensitivity at lower intensities (Gavriilidou et al. 2024) likely as a consequence of nGVS increasing the gain and sensitivity of vestibular afferents (Gavriilidou et al. 2024, see part 1). The effects of nGVS on posture is increasingly explored, as highlighted in a recent review that compiles studies investigating its influence on postural control (Xie et al. 2024). This study concludes that nGVS can enhance postural control through the stochastic resonance phenomenon when applied at subthreshold levels, with promising potential for vestibular rehabilitation (see Sect. "Therapeutic GVS: applications to central neurological and peripheral vestibular disorders").

#### Electrode configurations and postural response

As seen above, the most commonly used setup is the bilateral bipolar montage, inducing lateral body sway toward the anode (e.g. Britton et al. 1993; Fitzpatrick et al. 1994; Day et al. 1997; Séverac Cauquil et al. 2000; Cathers et al. 2005).

Other studies have investigated unilateral montages (see Sect. 1.3 Fig. 1B; Magnusson et al. 1990; Mihalik 1992), which produce a lateral sway toward the anodal electrode or away from the cathodal electrode, with equal amplitude. Later Séverac Cauquil et al. (1997) investigated anteroposterior and lateral sways evoked by monaural monopolar GVS first successively on a rocking platform then simultaneously on a force platform (Séverac Cauquil et al. 2000). They described an induced oblique, stereotyped body sway with the anteroposterior component comprising a forward or a backward deviation when the cathode or the anode was on the mastoid, respectively. The lateral component was twice as small in the monaural mode. The authors conclude that the classic binaural bipolar GVS could result from the addition of two complementary monaural stimulations (see Sect. 1.3 Fig. 2B, Séverac Cauquil et al. 2000).

This was confirmed using double monaural GVS, applying the same polarization on both mastoids (Fig. 2A) to successfully and consistently orientate the GVS response along the anteroposterior axis (Njiokiktjien and Folkerts 1971; Krizkova and Hlavačka 1994; Séverac Cauquil et al. 1998, 2000; Aoyama et al. 2015). Altogether, these studies suggest that the orientation of the response to GVS is a function of the imbalance between right and left vestibular polarization (Fitzpatrick and Day 2004). Scinicariello et al. (Scinicariello et al. 2002) observed that the sagittal sway caused by bilateral unipolar GVS is smaller in magnitude than the lateral sway induced by the bilateral bipolar configuration likely due to the distinct biomechanical properties of the body along the two axes.

Interestingly, further consideration of this concept led some authors to use a double monaural configuration applying opposite polarities to the mastoids. (Fig. 2B). Séverac Cauquil et al. (2000) demonstrated that this setup produces postural responses similar to these observed with classical binaural bipolar stimulation (see Fig. 1A), suggesting that the polarity of electrodes placed on the mastoids plays a determining role in the direction of the induced movement. Aoyama et al. (2015) reported that this type of double unilateral setup also induces a yaw movement rather than pure lateral sway. However, since they used a magnetic position sensor attached to the head without the use of a force platform, they were unable to capture full-body postural responses.

To conclude this section on postural responses to GVS, it is important to highlight that the main effect observed is a body sway toward the anodal side, emphasizing the crucial role of electrode polarity in determining the direction of the induced movement. However, the body sway response is not built on the sole vestibular information, it is shaped by the integration of multiple sensory modalities involved in postural control. In particular, both proprioceptive and visual information significantly modulate the amplitude and direction of body sway. The contribution of the vestibular system can be isolated by minimizing inputs from other modalities, for instance, by asking participants to close their eyes or by reducing proprioceptive feedback through changes in foot positioning. Ultimately, while the multisensory nature of postural responses adds complexity and limits the ability to isolate vestibular contributions, it also offers therapeutic advantages. Rehabilitation strategies can target and enhance other sensory pathways to compensate for vestibular deficits. This complexity contrasts with oculomotor responses to GVS, less influenced by the muscular proprioception modality since eyes are not submitted to gravity, and offer a more direct access to vestibular processing.

### Oculomotor responses to GVS

**Fig. 4:**
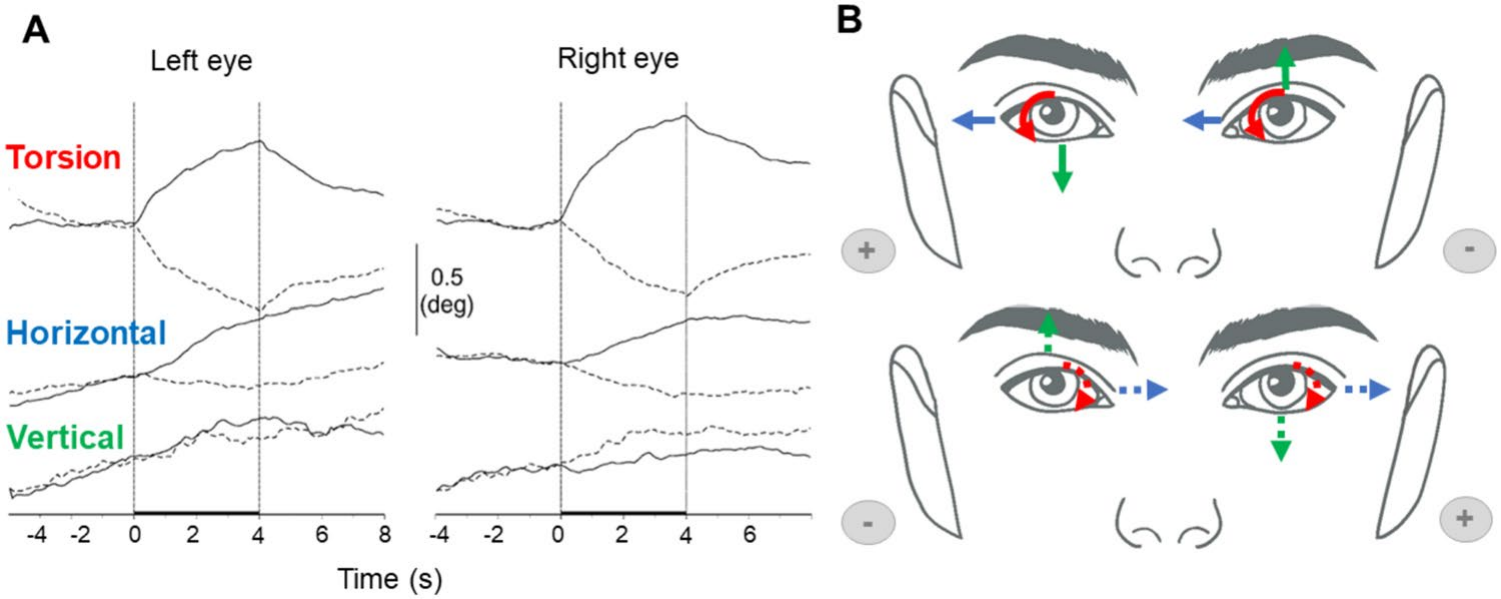
**A**, **B** Stereotypical ocular responses (torsion, horizontal deviation, and vertical divergence) induced by bipolar binaural GVS. **A** Traces showing grand average (n = 6) torsional, horizontal, and vertical position responses to 0.5 mA GVS, adapted from Séverac Cauquil et al. (2003). The graphs display the complete 12-s recording period, with dashed vertical lines indicating stimulus onset and offset. Positive values correspond to clockwise (torsional), rightward (horizontal), and upward (vertical) movements from the participant’s perspective. Solid lines represent cathode-left stimulation, while dotted lines correspond to cathode-right stimulation. **B** Representation of ocular responses induced by GVS in a binaural bipolar montage. The upper panel represents a configuration with the cathode (−) on the left, and the lower panel with the cathode (−) on the right. Ocular movements are color-coded: torsion (red), horizontal (blue), and vertical (green)

As for posture, most studies investigating ocular responses to GVS have used binaural bipolar electrode configuration (Zink et al. 1997, 1998; Watson et al. 1998a; MacDougall et al. 2002, 2003; Séverac Cauquil et al. 2003; Aw et al. 2006). The main ocular effect of GVS is torsional movement toward the anode, characterized by the upper poles of the eyes rotating away from the cathode (Zink et al. 1997, 1998; MacDougall et al. 2002, 2003; Séverac Cauquil et al. 2003; Aw et al. 2006). Horizontal eye movements are also observed, in the same direction as the torsion response: both eyes move away from the cathode and toward the anode (MacDougall et al. 2003; Séverac Cauquil et al. 2003; Jahn et al. 2003a, b; Aw et al. 2006). The least response is a skew deviation (or vertical divergence) characterized by hypertropia on the cathodal side and hypotropia in the anodal side (Séverac Cauquil et al. 2003; Aw et al. 2006). All ocular movements are illustrated in Fig. 4.

The mechanisms underlying torsional responses involve a complex interaction of vestibular structures. Some studies focused on utricular activation (Zink et al. 1997, 1998) while others highlighted the dominant role of the vertical semicircular canals (superior and posterior) in mediating torsional responses, whose combined activation amplifies torsion (Fitzpatrick and Day 2004; Aw et al. 2006). While the utricles play a significant role in enhancing torsion, the saccule contributes to a lesser extent, influencing fewer motoneurons involved in torsion and vertical movement (Suzuki et al. 1969; Isu et al. 2000; Fitzpatrick and Day 2004; Aw et al. 2006). Horizontal movements are primarily attributed to the activation of horizontal semicircular canals, which play a dominant role, with limited contributions from the utricular pathway. The amplitude of horizontal deviation is generally smaller than that of torsion likely due to potential antagonistic interaction between horizontal and torsional components (Fitzpatrick and Day 2004; Aw et al. 2006). Vertical movement results from the combined input of the superior and posterior SCCs, whose opposing vertical signals tend to partially cancel each other, leading to a reduced vertical response amplitude (Schmid-Priscoveanu et al. 2000; Kori et al. 2001; Aw et al. 2006). Altogether these results provide evidence that the ocular response to binaural bipolar GVS is consistent with an actual tilt of the head towards the anodal shoulder (Séverac Cauquil et al. 2003).

#### Factors modulating the ocular response

Current intensity plays the most significant role in shaping ocular responses: torsion, horizontal deviation, and skew deviation increase with intensity (Zink et al. 1998; Watson et al. 1998a; Séverac Cauquil et al. 2003; Aw et al. 2006). The intensity of the current also determines which vestibular structures are predominantly activated. Lower currents primarily engage the otolith pathways, eliciting smaller yet more stable responses (Zink et al. 1998; Aw et al. 2006). In contrast, higher intensities robustly stimulate the SCCs, leading to the amplification of torsion up to a ceiling value, after which nystagmus is triggered, characterized by slow phases directed toward the anode and quick phases beating toward the cathode (Cohen et al. 1964; Zink et al. 1998; Watson et al. 1998a; MacDougall et al. 2003; Jahn et al. 2003a, b; Aw et al. 2006). Horizontal nystagmus is most prominent at the onset and offset of stimulation (MacDougall et al. 2002) and arises from the activation of SCC afferents, driven by the rapid but short-lived responses of irregular fibers, which adapt quickly to sustained stimulation (Zink et al. 1998; Aw et al. 2006). This contrasts with ocular torsion, which is sustained by regular afferents with slower adaptation rates, ensuring prolonged responses during continuous stimulation (MacDougall et al. 2002).

In addition to current intensity, the type of stimulation montage also plays a key role in shaping ocular responses (Watson et al. 1998a; MacDougall et al. 2003). Unilateral stimulation (Fig. 1B) generates torsion directed away from the cathode, resembling the response observed with bilateral stimulation. However, bilateral stimulation further amplifies these effects, producing stronger torsion and nystagmus amplitudes. This enhancement is attributed to additive mechanisms in the vestibular nuclei, where inputs from both vestibular systems are linearly summed (Galiana et al. 1984; Watson et al. 1998a; MacDougall et al. 2003). However, oblique, diagonal body responses obtained with such configurations (Séverac Cauquil et al. 1998, 2000) suggest that a more complex eye response is triggered here, maybe difficult to catch with conventional eye-tracking devices.

Another important variable to consider is the presence or absence of a fixation point which is often necessary in studies involving eye-tracking. Several studies have compared ocular responses to GVS in complete darkness and in the presence of a fixation point (MacDougall et al. 2003; Jahn et al. 2003b). These studies consistently show that fixation suppresses horizontal and vertical nystagmus, leaving torsional components as the only measurable response. Notably, torsional responses remain stable and reproducible even in the presence of a fixation point (MacDougall et al. 2003; Séverac Cauquil et al. 2003; Curthoys and MacDougall 2012). In complete darkness, all response components, including torsion and nystagmus, are amplified, with stronger effects observed at higher current intensities (Jahn et al. 2003a, b; MacDougall et al. 2003). However, darkness also increases noise from uncontrolled eye movements, which can complicate data interpretation (Jahn et al. 2003b).

The ocular responses induced by GVS exhibit substantial variability among participants, particularly in response amplitudes. Significant differences have been observed in the amplitude of torsional, horizontal, and vertical eye movements across participants (MacDougall et al. 2002, 2003; Jahn et al. 2003b). Ratios of response components, such as torsional to horizontal movements, also vary a lot between individuals (MacDougall et al. 2002). Despite this inter-individual variability, responses within the same individual remain consistent across repeated trials and conditions (MacDougall et al. 2002). This consistency suggests that these differences may arise from stable anatomical or functional features unique to each participant, such as variations in the sensitivity of vestibular afferent subpopulations (Goldberg et al. 1984).

Among these inter-individual differences, certain group-based patterns have also been identified, particularly with respect to gender and age (Jahn et al. 2003a, b). Females tend to exhibit slightly larger torsion and nystagmus amplitudes compared to males (Jahn et al. 2003a). These differences may be attributed to anatomical factors, such as variations in skull structure that affect current delivery (Welgampola and Colebatch 2001, 2002). Regarding age-related differences, ocular responses peak in middle age (40–59 years), likely reflecting compensatory mechanisms such as increased vestibular sensitivity. Declines observed after 60 years are associated with vestibular degeneration, including the loss of irregular afferents and central pathways (Rosenhall 1973; Jahn et al. 2003a, 2003b).

On top of these measurements of GVS-induced eye movements, a recent study showed a beneficial effect of GVS on convergence, divergence, and stereopsis in both younger (20–25 years) and older subjects (40–60 years). This results, especially the fact that the improvement in near convergence occurs only for the youngest open promising perspective in the use of GVS for both re-adaptation and aging investigation (Xavier et al. 2023).

Although GVS-evoked eye movements provide a valuable window on vestibulo-ocular interactions, several methodological and interpretative limitations need to be taken into account.

One of the main difficulties lies in the very low amplitude of these responses—often limited to a few degrees or less—which makes them highly sensitive to noise. Accurate measurement therefore requires high resolution oculographic systems with sufficient sampling rates to capture these subtle displacements. As pointed out by Curthoys and MacDougall (2012), an adequate sampling rate is essential for detecting transient eye movements such as nystagmus. For example, in the study by Watson et al. (1998a, b), the relatively low sampling rate probably contributed to the failure to capture the rapid phases of nystagmus, underlining the importance of appropriate technical parameters for reliable data acquisition.

The role of intensity also warrants careful consideration. The appearance of nystagmus at higher intensities may not reflect a genuine vestibular response to GVS but rather a biomechanical limit of the oculomotor system. In this view, the more relevant indicators of vestibular activation in response to GVS are torsional eye movements, skew deviation and horizontal displacement (as described in Sect. "Oculomotor responses to GVS"). Moreover, the rationale for using higher intensities should be questioned. Several studies have used relatively high current intensities—up to 5 mA in Watson et al. (1998a, b) and MacDougall et al. (2002), and up to 7 mA in Zink et al. (1998)—to obtain these responses. However, such high intensities may be unnecessary to elicit reliable responses. For example, Séverac Cauquil et al. (2003) demonstrated that the ocular response to GVS could be obtained clearly and distinctly with intensities lower than 1 mA. The use of lower intensities not only reduces participant discomfort during GVS protocols. It would also enhance the ethical acceptability of GVS in both basic or clinical research, without compromising the detection of key oculomotor responses.

Most oculomotor studies have focused on bipolar binaural and monaural GVS setups (Zink et al. 1997, 1998; Watson et al. 1998a; MacDougall et al. 2002, 2003; Séverac Cauquil et al. 2003; Aw et al. 2006), leaving the effects of alternative montages—such as binaural monopolar configurations—largely unexplored. This gap in the literature prevents a full understanding of how the direction of the current and the placement of the electrodes influence oculomotor responses. Furthermore, although noisy GVS (nGVS) has been increasingly studied for its effects on posture, its impact on eye movements remains unknown.

### Cortical correlates of GVS

The cortical activation elicited by GVS has been well studied using neuroimaging tools, mainly magnetic resonance imaging (MRI), enabling a discussion about possible mechanisms of the vestibular processing and multisensory integration in humans.

The MRI environment is a very particular setup for studying vestibular stimulation, mainly because of magnetic vestibular stimulation (MVS) that results from the strong magnetic fields of the scanner, generated by the presence of a magnet, interacting with the inner ear. This phenomenon has been shown to cause neglect-like spatial attention biases in humans lying in a 3 T MRI (as well as vertigo and nystagmus effects in humans lying in a 7 T scanner (Roberts et al. 2011). Further investigation (Mian et al. 2013) deciphered how magnetic fields create these effects, indicating that the vertigo and nystagmus evoked by MVS probably share a common mechanism, involving Lorentz forces acting on the endolymph, stimulating the cups of the inner ear's semicircular canals. More recently, these conclusions have been nuanced by emphasizing that although electromagnetic induction could theoretically trigger vestibular responses, behavioral evidence supporting this hypothesis remains limited (Bouisset et al. 2024). Further research on the vestibular effects of variable magnetic fields is thus necessary to fully understand these phenomena.

While it is important to be aware of these phenomena, it is also crucial to keep in mind that they do not nullify the results obtained when doing acquisitions involving GVS during an MRI acquisition. Indeed, given that the magnet force does not change over time during the acquisition, effects related to Lorentz forces, in terms of cortical activations, should not show up on contrast maps generated under a comparison between conditions in an experimental design.

**Fig. 5:**
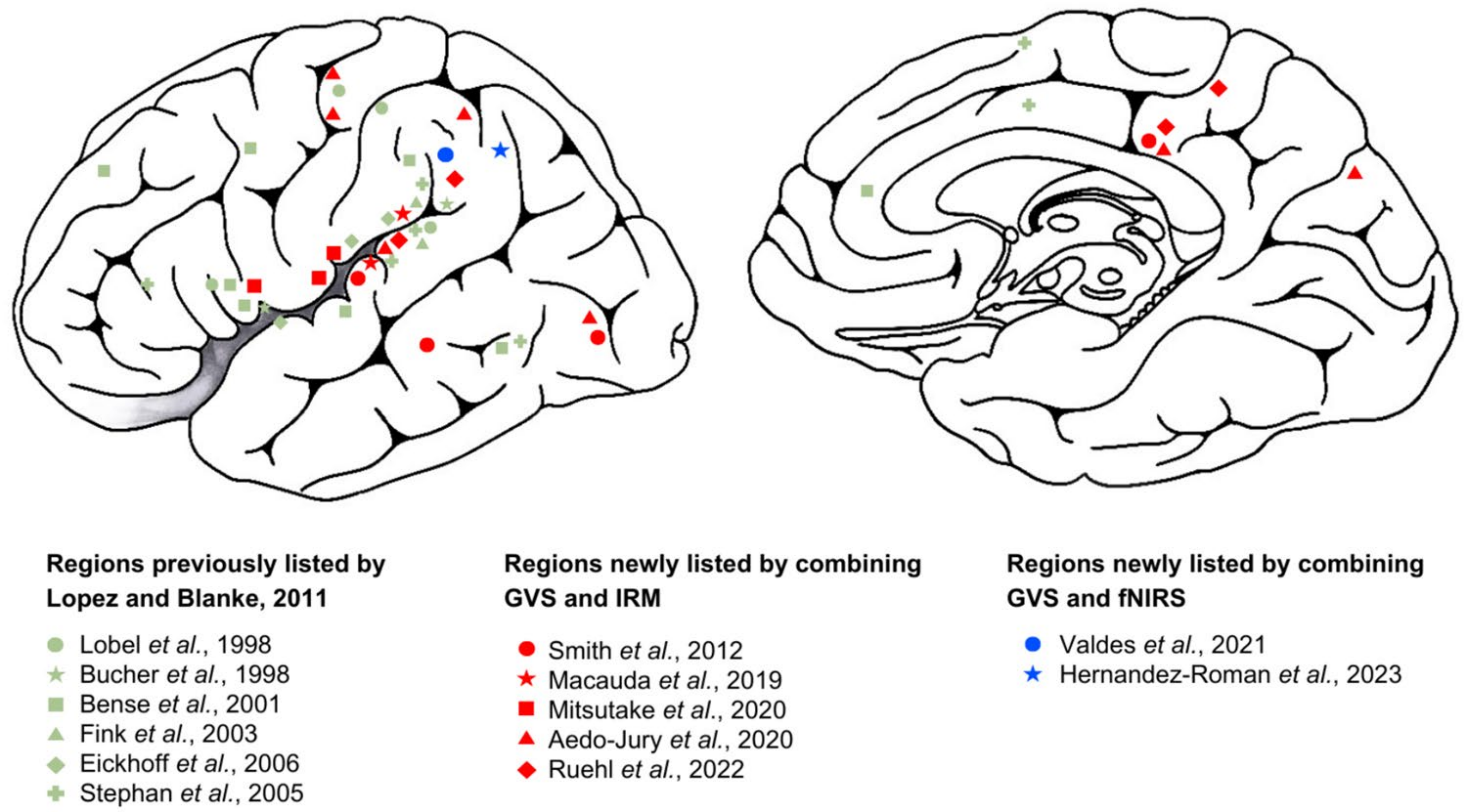
Vestibular sensitive cortical areas in humans revealed by neuroimagery, using MRI (red symbols) or fNIRS (blue symbols), combined with GVS. Previously known regions surveyed by Lopez and Blanke (2011) are shown in pale green. Inspired by Lopez and Blanke (2011)

Figure 5 indicates the GVS-activated cortical areas revealed by MRI and to a lesser extent functional near-infrared spectroscopy (fNIRS) studies. One of the first studies employing GVS during an MRI was reported to elicit bilateral activations of the middle (MI) and posterior insula (PI, demonstrating most prominent cortical response) as well as the transverse temporal gyrus (TTG) (Bucher et al. 1998). In the same year, another study found more regions activated during GVS, with activations of the temporoparietal junction (TPJ), intraparietal sulcus (IPS) and premotor regions of the frontal lobe (Lobel et al. 1998). Activations found in the central sulcus area, particularly in regions analogous to area 3a in humans, indicate that there might be direct vestibular projections to the somatosensory areas. Similarly, frontal activations, localized in area 6, suggest the presence of vestibular inputs in premotor regions. Negative BOLD responses in the transverse frontopolar gyrus also point toward inhibitory interactions, potentially engaged in compensatory mechanisms.

A more detailed mapping of cortical and subcortical activations elicited by GVS has been demonstrated by bilateral activations in the dorsolateral thalamus, the anterior insula, superior temporal gyrus (BA 22), inferior parietal lobule (BA 40) and the cerebellar hemispheres (Bense et al. 2001). Another pattern of activation was noted along the precentral gyrus (BA 6), middle frontal gyrus (BA 46/9), and the anterior cingulate gyrus (BA 32). In addition to these activations, there were signal decreases in the central sulcus, postcentral gyrus, visual cortex (outside BA 17), and the right precuneus. Negative BOLD responses are found in visual cortex, precuneus, and somatosensory areas, which is in line with previous reports of reciprocal inhibition between vestibular and visual modalities (Brandt et al. 1998). Such inhibition may reduce sensory conflict by gating out inputs from one modality in case of incongruent sensory information. Interestingly, the deactivation patterns are also modulated by stimulation frequency and current intensity, suggesting an adaptable neural mechanism that tunes itself to the changing sensory demand.

Studies using alternating current stimulation (AC-GVS) and direct current stimulation (DC-GVS) also demonstrated differences in activation patterns, suggesting that differences in stimulation parameters have an impact on cortical responses (Stephan et al. 2005). Studies of AC-GVS (Lobel et al. 1998) demonstrated activations in the temporoparietal junction and anterior intraparietal sulcus, whilst studies of DC-GVS (Bense et al. 2001) located the activations to the posterior insula and retroinsular regions. These findings point out the importance of stimulation characteristics in modulating the neural circuitry engaged during GVS. As seen in Sect. 1.4, DC current provides a directional, oriented although artificial input, whereas AC is supposed to enhance the global vestibular sensitivity.

Further work has continually supported hemispheric asymmetry in vestibular processing, with right-hemispheric dominance being a finding that is proving robust (Dieterich et al. 2003; Eickhoff et al. 2006; Lopez et al. 2012). Right-anodal (left-cathodal) GVS induced bilateral significant activation of the posterior parietal operculum (OP2), the anterior insula, and subcentral gyrus (Eickhoff et al. 2006). The OP2 region is considered the human equivalent of PIVC, being an essential site of convergence of the vestibular inputs. However, only the right side responded consistently to GVS regardless of stimulus polarity, supporting the hypothesis of a dominant right hemisphere for vestibular processing.

These GVS activations of OP2 were later confirmed (Macauda et al. 2019) along with evidence of PIC activations, which is in line with previous vestibular neuroimaging meta-analyses (zu Eulenburg et al. 2012; Lopez et al. 2012). These findings also emphasize the dominance of the right hemisphere for vestibular functions (Dieterich et al. 2003) and point to functional and anatomical connectivity between the PIC and the visual area of the cingulate sulcus (CSv), an area regularly activated by optic flow stimuli (Cardin and Smith 2010; Smith et al. 2018). Additionally, the GVS activates the bilateral middle cingulate sulcus, further implicating the CSv area in the integration of vestibular and visual information.

Based on this knowledge, the use of different GVS setups (see Sect. 1.4), allowing the dissociation between lateral (Lat) and anteroposterior (AP) stimulation, showed a consistent activation for lateral GVS of PIVC, 2v, 3aNv, hMT+, and CSv, whereas AP GVS showed a stronger response in V6 and VIP (Aedo-Jury et al. 2020). Associated connectivity analyses revealed dissociated processing networks under these conditions (Aedo-Jury et al. 2020). For example, AP GVS has been associated with increased connectivity between V6, CSv, hMT+, and PIC, whereas Lat GVS has been reported to activate primarily VIP, CSv, hMT+, and PIC. The strength of this activation for a wide range of stimulation parameters (Smith et al. 2012) suggests that these regions participate in the integration of vestibular and visual information.

Very recently, responses to GVS across egomotion hubs, extending to areas including CSv, PcM/pCi, VPS, h7a, has been described, further extending the role of vestibular stimulation in engaging multisensory and motor-related areas (Ruehl et al. 2022). Functional connectivity analyses showed that, under GVS, CSv shifted from visual-motion areas to the egomotion hubs, including SEF and OP2, supporting its role as a mediating hub between visual perception, motor control, and vestibular navigation.

Complementing these MRI studies, Hernández-Román et al. (2023) identified overlapping regions of the vestibular cortex (temporal and posterior parietal areas) using fNIRS, with GVS-induced increases in HbO_2_ concentrations particularly pronounced in the posterior parietal region. However, due to the limitations of fNIRS, responses in deeper structures like the insular cortex were not measurable. These findings are consistent with prior functional magnetic resonance imaging (fMRI) studies (e.g. Mitsutake et al. 2020), which reported increased activation in the parietal operculum, central operculum, and the opercular part of the inferior frontal gyrus during GVS. A previous study by Valdés et al. (2021) using fNIRS found that subthreshold noisy GVS (nGVS) led to a significant increase in deoxygenated hemoglobin (HbR) concentration in the left supramarginal gyrus (BA40), a key vestibular processing area, with a similar but non-significant trend in the right hemisphere.

MRI and fNIRS offer complementary insights on vestibular processing, each method showing advantages and limitations impacting the activation patterns revealed. MRI gives an overview of the vestibular network, with activations in both cortical and subcortical locations, including thalamus, insula, cerebellum, and visual motion-sensitive areas (e.g. hMT+, V6, and CSv). However, its use for vestibular studies is constrained by the supine position of subjects in the scanner, this probably preventing most of the perceptual response from arising. In addition, the strong static magnetic field produces Magnetic Vestibular Stimulation (MVS), which may interfere with GVS responses, even though this phenomenon has been demonstrated to cause neglect-type spatial attention biases (Roberts et al. 2011). Moreover, this does not affect the results of fMRI studies using contrasts between conditions, as MVS remains constant across all conditions. On the other hand, fNIRS is better suited for experiments involving seated subjects and natural head movements. It has higher temporal resolution than fMRI but is constrained by shallow penetration depth, limiting measurements to deeper cortical regions (e.g. the temporoparietal and posterior parietal cortices, the supramarginal gyrus, the opercular cortex). fNIRS is therefore unable to record subcortical activations and is less useful to map the entire extent of the vestibular network. These methodological variations enable fMRI to provide a more thorough view of cortical and subcortical vestibular processing, while fNIRS allows investigations under more ecological conditions. The choice of method depends on the question addressed, with fMRI being ideally suited to mapping deep brain structures and fNIRS offering benefits when protocols require naturalistic postures and movements.

Although GVS-induced neural activations have been broadly mapped by MRI and, more recently, fNIRS studies, crucial questions remain as to the precise roles of these vestibular-responsive regions. The strong dominance of the right hemisphere in vestibular processing suggests lateralized specialization, but the extent to which this asymmetry affects behavior and perception remains unclear. In addition, the interaction between vestibular, visual and somatosensory networks is still an unresolved issue, particularly in ecologically valid environments that allow natural head and body movements.

Knowing the limitations of MRI and fNIRS tools, future research should also focus on integrating these neuroimaging methods with electrophysiological techniques and behavioral paradigms to provide a more comprehensive understanding of vestibular processing and its multisensory integration.

### Perceptual effects of GVS

While GVS has been shown to influence broad cognitive processes, including attention, motivation and decision-making, this review focuses primarily on its effects on somatic and perceptual aspects. A comprehensive discussion of cognitive effects is beyond the scope of this review and can be found in recent articles (Preuss et al. 2017; Blini et al. 2018, 2020; De Maio et al. 2021).

#### Self-motion perception

Illusory self-motion perception induced by GVS was reported in the earliest publications as vertigo (e.g. Hitzig 1874), however few studies objectively quantified this perceptive output. Later it has been related that binaural bipolar GVS can induce small (~ 5 to 15°) oscillations sensation of the body around a mid-sagittal axis (roll), in seated and supine position, (Lobel et al. 1998). The oscillations sensation being weaker when performed during fMRI sessions, possibly due to the influence of the scanning environment on sensory processing. Larger rotation sensations (90°–360°) around the same axis were described using stronger binaural bipolar GVS that also triggered discomfort associated with pricking sensation in or around the site of the electrode (Bense et al. 2001). The same binaural bipolar GVS configuration deviates towards the cathode side the self-motion perception during the course of visually induced vection (Lepecq et al. 2006).

Most importantly, self-motion illusion occurs when body sway is prevented otherwise subjects report their actual body sway response in the opposite direction (Fitzpatrick et al. 1994). Later research showed additive effects where natural body rotation was in the same direction as GVS and enhanced perceived motion while incongruent stimuli reduced the effects, showing that the vestibular system integrates the different sensory inputs in order to modify perception (Fitzpatrick et al. 2002). In addition, a study demonstrated that combining natural rotation with a well-calibrated GVS signal can effectively cancel the sensation of rotation in roll, leading instead to the illusion of linear translation (Schneider et al. 2009). This effect, predicted by SCC-otolith interaction models, suggests that GVS can be used to induce virtual linear translation without visual stimuli or centrifuge, which has potential applications in vestibular testing and motion simulation. Self-adjusted amplitude and phase of GVS was able to minimize the roll sensation during actual whole-body rotation, generating a residual linear translation possibly due to gravitoinertial force and/or otolithic GVS activation. Furthermore, GVS has been shown to systematically amplify or attenuate perceptions of roll tilt, depending on the waveform applied (Allred et al. 2024). A novel computational model now allows prediction of 6 degrees-of-freedom self-motion and orientation perceptions under GVS, by integrating vestibular afferent neuron dynamics with a central processing observer model.

St George et al. (2011) applied on subjects head pitched forward a trapezoidal prolonged GVS that evoked a sensation of rotation towards the cathodal side, about a vertical axis akin, but lasting longer than the one obtained from kinetic, whole body rotation on a rotating platform. Rather interestingly, the highest stimulus intensity led to longer adaptation periods, thus possibly indicating that the brain dynamically readjusts the vestibular input, the duration of the increase of intensity being the same, the slope being steepest for the highest intensity. Further studies are needed to disentangle the intensity effect from the velocity one. Also GVS-induced whole-body rotation illusions are dependent upon the orientation of the galvanic vector relative to head posture (St George and Fitzpatrick 2011).

The frequency of sinusoidal GVS significantly influences the type and strength of the perceptual effects. The optimal range for the induction of self-motion illusions is below 1 Hz, with the strongest sensations reported between 1 and 2 Hz (Stephan et al. 2005). Here, subjects verbally reported consistent sensations of rotation again mainly on the roll axis.

Even fewer studies investigated the GVS-induced motion illusion for other electrode configurations other than the usual binaural bipolar mode. Aoyama et al., (Aoyama et al. 2015) attempted to compare self-rotation sensations by conventional binaural bipolar (two-pole) GVS, binaural monopolar (four-pole), and binaural bipolar four-pole GVS (see Figs. 1A, 2). The participants related that roll, pitch and yaw rotation sensations are evoked by the 3 GVS configurations, respectively. However the subjects were standing eyes closed and free to move, hence they most likely reported their actual body sway rather than the self-motion illusion (Camis 1930; Fitzpatrick et al. 1994; Wardman et al. 2003b).

#### Visuo-vestibular interactions in percept

Recent studies show that visual input does not suppress GVS-induced egomotion. In the Ruehl et al.'s (2022) MRI experiment, in particular, all subjects verbally reported egomotion sensation on eyes open (illusion intensity 6.13 ± 1.99/10; mean ± SD) and eyes closed (5.84 ± 1.87/10) condition. Additionally, it was found that both GVS and optokinetic stimulation (OKS) independently induce biases in the perception of vertical, and these biases follow an exponential time course. When applied together, the effects of GVS and OKS combine linearly, reinforcing the idea that optokinetic and artificial vestibular cues complement each other in estimating head angular velocity (Niehof et al. 2019).

GVS also affects the sense of vision and such modulation of apparent visual scenes by GVS has been well studied over the last few years, for example on subjective vertical (Zink et al. 1997, 1998; Tardy-Gervet and Séverac-Cauquil 1998). The polarity of stimulation corresponds with reported perceived line tilts, pointing to interactions between vestibular input and the process of vision. Torsion effects during prolonged GVS caused dissociations between eye movements and perception of tilt pointing to multi-dimensional effects on spatial orientation during GVS (Watson et al. 1998a), but not only since Mars et al. (2001), evidenced residual subjective vertical deviation in eyes closed somatosensory conditions. These authors went further by showing the lack of correlation between GVS-induced subjective vertical and subjective body orientation deviation, evidencing that different central processes elaborating different references are involved (Mars et al. 2005). Extending these findings, further research demonstrated that GVS reduced visual vertical errors in stroke patients (Lopez 2016), which suggests the potential of GVS as a treatment for balance and spatial perception deficits.

#### GVS effects on body and space representation

GVS can influence the apparent direction of motion, generally causing lateral deviations of vection trajectories. GVS deviated subjects' reaching movements towards the anodal side in complete darkness (Bresciani et al. 2002), which suggests that GVS affects spatial relationship between the target and the hand. These findings suggest complex interactions between the vestibular inputs, head orientation, and perceived motion.

Binaural bipolar GVS induced symmetrical bisection bias towards the anode side, right or left, in both near and far space (Ferrè et al. 2013). On the contrary a polarity effect was found on somatosensory sensitivity on both hands that was enhanced by anode-left cathode-right GVS, and not the other polarity (Ferrè et al. 2015). By using the rubber hand illusion paradigm, the same group suggested that GVS could modify bodily awareness (Ferrè et al. 2015). This stimulation strengthened the visual dominance of body ownership and perception in RHI. The findings are crucial, as they outline the great involvement of the vestibular system in maintaining body awareness and integration of sensory information.

As an aeronautical application, GVS has been proposed as a training tool for pilots. A study evaluated the use of GVS to generate common flight illusions in virtual reality (VR), such as the somatogravic and Coriolis illusions, by intentionally mismatching GVS applications with the visual scene. This approach could help prepare pilots for vestibular disturbances encountered in real flight (Pradhan et al. 2022).

Research on GVS has demonstrated its significant impact on motion perception and spatial cognition. However, although the effects induced by GVS on body and space representation are becoming better documented, there remain significant gaps in our understanding of the underlying mechanisms: the precise neural pathways by which GVS modulates vestibular processing are not entirely elucidated and further research is needed to fully understand how different electrode configurations and stimulation parameters influence the variability of perceptual effects. The integration of GVS with other sensory modalities also requires further exploration to better understand the interaction between these inputs in real-world contexts. In addition, while GVS seems to be a promising tool in applications such as motion simulation and vestibular rehabilitation, its clinical implications have yet to be fully realized, particularly in patients with complex vestibular disorders.

## GVS in clinical research: applications to vestibular and central neurological disorders

Many pathologies of the central nervous system (e.g. central positional vertigo, vestibular migraine, ischemia in the vertebral-basilar artery system, multiple sclerosis) and the peripheral vestibular system (e.g. vestibular neuronitis, Ménière’s syndrome, benign paroxysmal) can cause dizziness, vertigo and balance disorders (Salvinelli et al. 2003). Most of them are described in the International Classification of Vestibular Disorders (ICVD) emanating from the Barany Society (Bisdorff et al. 2009).

The medical history and clinical examination are very important in the diagnostic process (Salvinelli et al. 2003) and should be accompanied by a series of tests and possibly medical imaging. In routine clinical practice, patients with dizziness can be evaluated using video head impulse tests (Halmagyi et al. 2017), videonystagmography (Falls 2019), VEMPs (Rosengren et al. 2009), and posturography (Falls 2019). They may also receive prescriptions for medical imaging, to be performed urgently or not, like MRI et CT-scan although it is necessary to be wary of the predictive value of imaging with specificity and sensitivity not always optimal (Shah et al. 2023).

Generally, the treatment of dizziness can be divided into three categories (Salvinelli et al. 2003), although these categories may be combined depending on the pathologies: medications (specific and nonspecific), surgery (conservative and destructive), and rehabilitation (vestibular rehabilitation and classic reeducation).

To decrease patients’ symptoms, it is essential to accurately diagnose the problem, and then select the optimal treatment. In that respect, GVS appeared very quickly as a potential tool. The first insights about the clinical usefulness of GVS date back over a century. Babinski (1901) observed that patients with complete bilateral deafness did not respond to GVS in the same way as healthy subjects. Indeed, healthy subjects experienced vertigo at low stimulation intensities (1–2 mA), compared to profoundly deaf patients, for whom very high intensities (10–12 mA) were required to elicit even slight postural reactions. A few decades later, these results were replicated in patients with areflexic labyrinths to caloric stimulation, no responding to GVS as high as 10–20 mA when the anode or cathode was positioned on the mastoid of the pathological ear (Blonder and Davis 1936; Watanabe et al. 1985). On oculomotricity, Pfaltz observed that a high-intensity galvanic current (15 mA) applied to an ear with peripheral nerve impairment did not generate any nystagmus (Pfaltz and Richter 2007). Later, Tokita et al. (1989) hypothesized that observing GVS-induced nystagmus and spinal reflexes could enable clinicians to differentiate between otolithic lesions and canal lesions. A diagnostic tool based on low-intensity GVS was developed and used on retro-labyrinthine disorders (Watanabe et al. 1989). Then, the interest of GVS as a rehabilitation instrument for central and peripheral neurological pathologies (Parkinson's disease, unilateral vestibular disorders, stroke) has been investigated (Fujita et al. 1993; Pastor et al. 1993; Mbongo et al. 2005; Saj et al. 2006), giving rise to a still ongoing interest for GVS in clinics.

In recent years, there has been a growing interest in vestibular galvanic stimulation for both the investigation and treatment of dizziness and balance disorders (of both central and peripheral origin), accompanied by a significant increase of publications. Here, we follow our review of the recent literature on these various perspectives.

### Galvanic vestibular evoked potentials for exploration and diagnosis

At the end of the twentieth century, Watson and his collaborators published various works on the potentials evoked by GVS (Watson and Colebatch 1997, 1998; Watson et al. 1998b). They are the first to explore this type of induced response in humans. The objective of this stimulation technique was to analyze variations in the EMG of different body muscles (soleus, sternocleidomastoid and ocular muscles) in response to GVS.

By stimulating using binaural bipolar montage electrodes on both mastoid processes, using a 20 ms, 4 mA stimulus at 3 times per second over 256 trials, they obtained an evoked potential in the soleus (Watson and Colebatch 1997, 1998). The latency of this evoked potential depended on the type of montage used (binaural bipolar, monaural anodal, monaural cathodal), but generally it appeared between 53 and 61 ms, and ended between 94 and 102 ms for a duration of 39–42 ms (Watson and Colebatch 1997). They concluded that vestibular activation by GVS generates the same responses in the soleus as sound clicks does (Watson and Colebatch 1998). These evoked potentials disappeared after vestibular nerve section (Watson and Colebatch 1997).

Finally, Cunha et al. (2014) examined the vestibular evoked myogenic potentials (VEMPs) induced by binaural galvanic stimulation and confirmed the presence of biphasic responses in the soleus muscle, consisting of a short latency (SL) component at 54 ms and a medium latency (ML) component at 112 ms (Cunha et al. 2014). The SL component would be associated with the function of the otoliths (saccule and utricle), while the ML component would reflect the activation of the semicircular canals (Cathers et al. 2005). This study highlighted the reproducibility of VEMP responses and their relevance for assessing vestibulospinal function. Based on the knowledge of these potentials evoked by GVS, a recent study used these evoked potentials to assess the effects of sleep deprivation on cognitive and sensorimotor functions, particularly balance (Copeland et al. 2024).

**Fig. 6:**
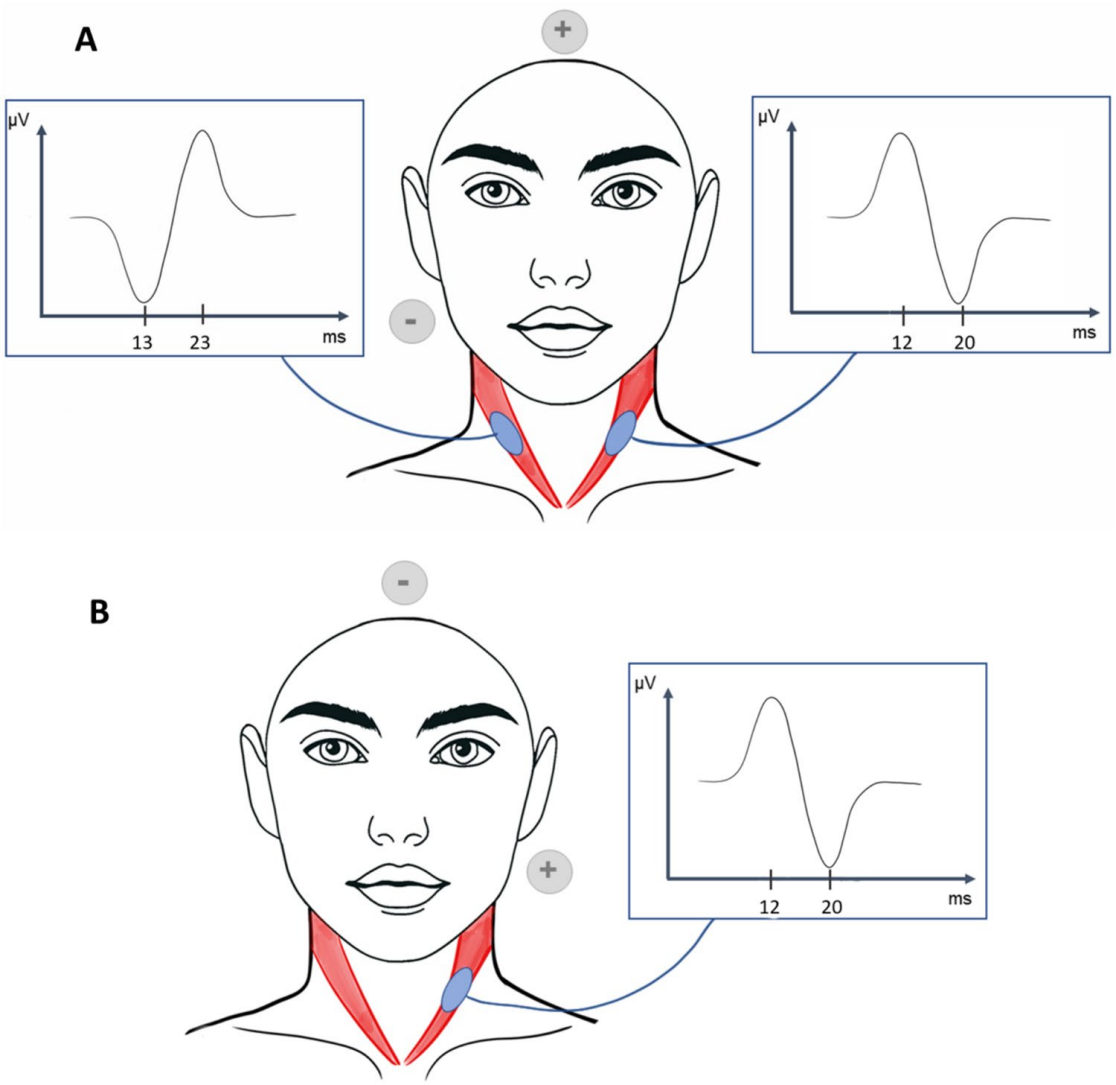
Latencies of cervical vestibular evoked myogenic potentials generated by GVS, **A** unilateral galvanic stimulation with cathode on mastoïd; **B** unilateral galvanic stimulation with anode on mastoid

Similarly, they stimulated with a 2 ms pulse at an intensity of 4–5 mA at a rate of 5 stimulations per second (256 repetitions) to record an evoked potential in the ipsilateral sternocleidomastoid muscle (Fig. 6) under both normal and pathological conditions (Watson et al. 1998b; Watson and Colebatch 1998). Unilateral galvanic stimulation revealed the p13/n23 response (positivity at 13 ms, and negativity at 23 ms) to be solely generated by the cathode (Watson et al. 1998b). A biphasic response, starting with a surface negativity (n12p20), was observed contralateral to the cathode in all subjects and was produced by both the cathode on the contralateral side and the anode on the ipsilateral side (Watson et al. 1998b).

Again, unilateral vestibular nerve section abolishes these evoked potentials recorded in the ipsilateral and contralateral SCM (Watson et al. 1998b).

**Fig. 7:**
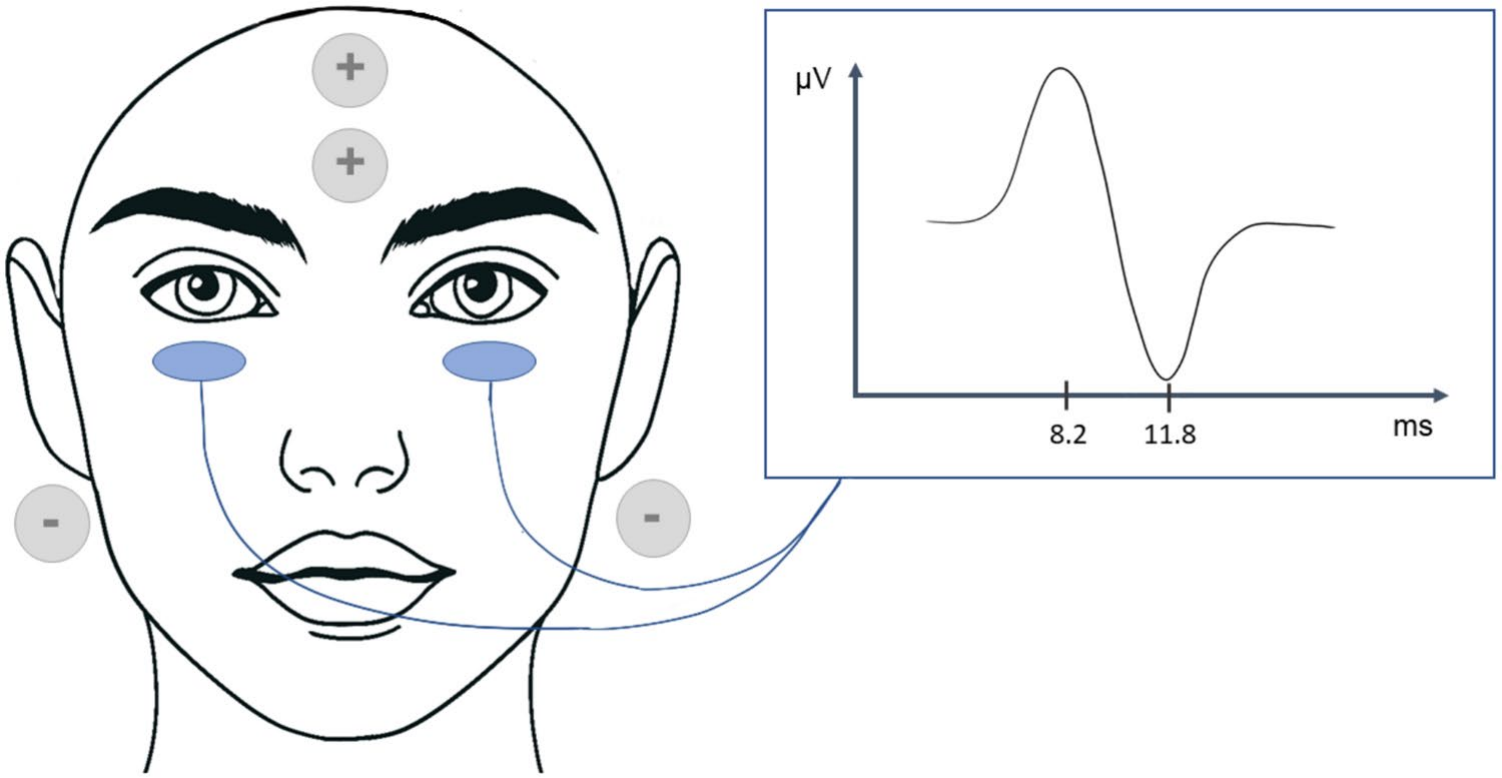
Latencies of ocular vestibular evoked myogenic potentials generated by GVS

In addition to cervical VEMPs and soleus VEMPs, oculomotor responses can be observed during vestibular stimulation (Fig. 7). The oculomotor responses generated by GVSare described in greater detail earlier. Here, we only describe the onset latencies of these responses. The latencies of the ocular vestibular evoked myogenic potential generated by GVS are 8.2 ± 0.6 ms for the negativity and 11.8 ± 0.6 ms for the positivity with a direct 5 mA current for 1.0 ms (Cheng et al. 2009). The nI-pI interval was 3.6 ± 0.4 ms, and the nI-pI amplitude was 13.7 ± 5.4 µV (Cheng et al. 2009).

Using stimulation parameters similar to Watson and Colebatch (1998), research has since been conducted to establish normative values for vestibulocollic evoked potentials generated by GVS, as well as to analyze the effect of age on these evoked potentials (Welgampola and Colebatch 2001; Avcı et al. 2022). It appears that the amplitude of the p13/n23 complex decreases with increasing age (Welgampola and Colebatch 2001), while the latency of the complex's onset seems to increase with age (Avcı et al. 2022). We can imagine that these normative data may assist us in diagnosing retrolabyrinthine lesions (Avcı et al. 2022). Consistently with studies dealing with GVS site of action (see Sect. «The different GVS configurations»), biphasic EMG responses still exist in the presence of labyrinthine lesions, even on the affected side (Murofushi et al. 2002). In contrast, with retrolabyrinthine lesions, we observe no response or a diminished response on the affected side (Murofushi et al. 2002). Therefore, myogenic responses evoked by galvanic stimulation on the SCM may be useful in the differential diagnosis between labyrinthine and retrolabyrinthine lesions, especially in patients with an absence of click-evoked vestibulo-collic reflexes (Murofushi et al. 2002).

The presence of these potentials evoked by GVS can be a major asset in clinical exploration of vestibular disorders, such as Ménières’s disease and idiopathic sudden hearing loss.

Regarding GVS-VEMPs in the electrophysiological assessment of Ménière's disease, it seems that their use may not be of significant interest, as the results do not show a difference between a healthy ear and a pathological ear in the acute phase of the disease (Chang et al. 2017). This is confirmed by a subsequent study that did not find differences in the results of galvanic-VEMPs between a group of patients with unilateral Ménière's disease and a group of healthy subjects (Cheng et al. 2022).

Galvanic-VEMPs can also help identify the location of a lesion in cases of idiopathic sudden hearing loss accompanied by vertigo and clarify the extent of vestibular lesions (Iwasaki et al. 2005). In cases of idiopathic sudden hearing loss of purely labyrinthine origin, we are expected to find normal responses in galvanic-VEMPs (Iwasaki et al. 2005), whereas abnormalities are highly likely to be found in click-VEMPs (77%) and caloric tests (45%). The combination of VEMPs (air-conducted sound and galvanic) and caloric tests is useful for evaluating vestibular functions in idiopathic sudden hearing loss with vertigo, as the extent of vestibular abnormalities is well correlated with auditory outcomes (Iwasaki et al. 2005).

### Therapeutic GVS: applications to central neurological and peripheral vestibular disorders

Whereas the scientific community studying the therapeutic effects of GVS had primarily focused on central neurological pathologies (e.g. stroke, Parkinson’s disease, spinal cord injuries), it is now obvious that a shift has occurred. Clinical studies that utilized GVS in the treatment of vertigo and balance disorders (both central and peripheral) were selected.

Table 1 presents the methodology and results of studies regarding central disorders and pathologies as well as peripheral vestibular dysfunctions. All studies cited used binaural (and bipolar if not specified) GVS. To simplify the reading of this section, the table below presents the main results of recent original studies, followed in the text by a comparison and discussion with previous original publications and reviews.

**Table 1:** Summary of clinical studies using GVS in central neurological disorders and peripheral vestibular dysfunctions

References	Design	Population	Characterization of GVS	Outcomes measures
Tohyama et al. (2021)	Single-blind study	Stroke n = 24 patients (10 left hemispheric lesion/14 right hemispheric lesion) n = 9 healthy control group	Binaural GVS in three conditions: contralesional-anodal and ipsilesional-cathodal vestibular stimulation (ipsiVS), ipsilesional-anodal and contralesional-cathodal vestibular stimulation (contraVS), and no stimulation 1.5 mA electric current	Standing posture on force platforms Assessment of subjective visual vertical
	*Results*: SVV was modulated by reversing the polarity of GVS in all the groups when the cathodal stimulus side was either ipsilateral or contralateral to the lesion while the ipsilesional-cathodal vestibular stimulation reduced weight-bearing asymmetry in only the patients with a right hemisphere lesion			
Tomioka et al. (2022)	Clinical trial	Stroke n = 20 unilateral supratentorial lesion (10 left hemispheric lesion/10 right hemispheric lesion)	Binaural GVS (three stimulation patterns: right anode/left cathode, left anode/right cathode, sham) 1.5 mA electric current	Subjective visual vertical Center of gravity positions in the sitting posture
	*Results*: instantaneous modification of subjective visual vertical and center of pressure in the sitting position for different setups compared to sham stimulation. No significant difference in outcomes between the two binaural stimulation patterns			
Horikawa et al. (2024)	Assessor blinded, randomized, cross-controlled trial	Post-stroke left hemiplegia n = 22	Binaural GVS (right anode/left cathode, left anode/right cathode) 3.0 mA electric current	Assessment of postural righting reaction (center of pressure measurement, analyze joint angles of neck, trunk and leg during postural straightening reactions)
	*Results*: GVS with anodal stimulation on the paralyzed side could promote righting reactions in patients with post-stroke hemiplegia			
Lee et al. (2021b)	Clinical trial	Parkinson’s disease n = 18 parkinson’s disease group n = 20 healthy participants	Binaural GVS Intensity: 90% of the determined threshold value nine stimulation conditions: off-stimulation, Random Noise RN (4–200 Hz), Multisine Stimuli (ms)-θ (4–8 Hz), ms-α (8–13 Hz), ms-β (13–30 Hz), ms-γ (30–50 Hz), ms-h1 (50–100 Hz), ms-h2 (100–150 Hz), and ms-h3 (150–200 Hz)	Simple reaction time task to visual stimulation
	*Results*: using band-limited ms-GVS, the GVS frequency for the best response time varied considerably across participants and was > 30 Hz for half of the Parkinson Disease with off medication levodopa patients			
Duncan et al. (2024)	Clinical trial	Parkinson’s disease n = 17	Binaural GVS (right cathode/left anode, and sham stimulation) 0.25 to 0.35 mA electrical current	Finger and foot taping task EEG (spectral power, mu-rhythm range)
	*Results*: compared to the sham, GVS was positively correlated with higher amplitudes during the late and movement phases of the Bereitschaftspotential, as well as a more pronounced reduction in spectral power within the mu-rhythm range during finger-tapping			
Gutkovich et al. (2022)	Prospective, single-blind, randomized controlled trial	Seasickness n = 15 treatment group (GVS coupled with inverse phase rotatory chair impulse in sinusoidal harmonic acceleration protocol) n = 15 control group (rotatory chair impulse in sinusoidal harmonic acceleration protocol without GVS)	Binaural sinusoidal GVS modulated with noise The electrical current intensity not exceeding 3 mA Initially, stimulation was given at 1 mA, with increments of 0.5 mA until the subject experienced discomfort. The intensity was then reduced to 75% of this level	Vestibular time constant Anti-motion sickness drug consumption - Wiker seasickness questionnaire
	*Results*: the GVS combined with rotatory chair treatment reduced the incidence of severe seasickness. Three months after the intervention, the treatment group showed a decrease in both the number of visits to motion sickness clinics and the consumption of anti-motion sickness medications compared to the control group. Additionally, there was a significant reduction in the vestibular time constant (Tc) in the treatment group			
Ceylan et al. (2021)	Clinical trial with control group	Unilateral peripheral vestibular pathology n = 42 study group (GVS + vestibular rehabilitation) n = 35 control group (vestibular rehabilitation)	Binaural GVS bipolar stimulation 1, 2, 3, 4 and 5 mA electric current Cathode on the hypofunctional ear, and anode on the other ear	Sensory organization testing in computerized dynamic posturography Visual analog scale for assessing discomforts (VAS)
	*Results*: better balance score (sensory organization testing) and VAS discomfort in study group compared to control group			
Nguyen et al. (2023)	Clinical trial	Vestibulopathy and cerebellar disorders n = 31 (18 unilateral or bilateral vestibulopathy/13 cerebellar ataxia)	Binaural GVS Three parameters, waveform (sinusoidal, direct current [DC], and noisy), amplitude (0.4, 0.8, and 1.2 mA), and duration of stimulation (5 and 30 min), resulting in a total of 18 GVS intervention modes as input variables	Visual Analogic Scale for clinical vertigo and gait assessments Activities-specific Balance Confidence Scale (ABC) Scale for Assessment and Rating of Ataxia (SARA)
	*Results*: patients with unilateral vestibulopathy experienced the most favorable results with either noisy or sinusoidal GVS at 0.4 mA amplitude for 30 min, followed by DC GVS at 0.8 mA amplitude for 5 min. Noisy GVS at 0.8 or 0.4 mA amplitude for 30 min demonstrated the most beneficial effects in patients with bilateral vestibulopathy. For patients with cerebellar ataxia, the optimal choices were noisy GVS with 0.8 or 0.4 mA amplitude for 5 or 30 min			

#### Stroke

##### Postural asymmetry and pushing behavior

A study demonstrated that the administration of binaural sinusoidal GVS at 2 mA, with the anode applied to the ipsilesional CVPI, significantly increased the reaction speed of subjects to balance disturbances (Babyar et al. 2018). This stimulation accelerated the velocity of the center of pressure displacement, thereby improving the patient's lateropulsion (Babyar et al. 2018) compared to when subjects were subjected to a placebo condition under the same experimental setup. Trunk inclinometry measurements also confirmed these results (Babyar et al. 2018).

Ipsilesional cathodal vestibular stimulation (binaural GVS) was found to be effective in reducing weight-bearing asymmetry, but this effect was only observed in patients with right hemisphere lesions (Tohyama et al. 2021). Bonan et al.’s (2016) study supports these observations and provides additional details: GVS (binaural of 35 s and 2 mA) appears to reduce postural asymmetry in both patients with right and left brain lesions. However, in the group with right hemisphere lesions, the velocity of center of pressure displacement was significantly higher when left cathodal GVS was applied, compared to the control group and the group with left hemisphere lesions (2.8 and 2.4 times higher, respectively, with a statistically significant difference). Notably, these findings did not indicate a specific involvement of CVPI lesions, as no significant difference in postural asymmetry improvement was observed between patients with or without lesions in this area (Bonan et al. 2016).

Moreover, GVS with cathodal stimulation applied to the lesional side could promote righting reactions in patients with post-stroke hemiplegia (Horikawa et al. 2024). Immediate modifications of center of pressure in the sitting position were also reported for different stimulation parameters compared to sham stimulation. However, no significant difference was noted between the two tested binaural stimulation modes, namely, right anode/left cathode and right cathode/left anode (Tomioka et al. 2022).

Regarding pushing behavior, the results indicate that a single GVS session (20 min of stimulation, anode on the ipsilesional side/cathode on the contralesional side, at a fluctuating intensity of 1 mA, 1.25 mA, 1.5 mA, 1.75 mA, and 2 mA) has no significant effect. No improvement in the scale for contraversive pushing (SCP) score was observed between the beginning and the end of the session, a finding similar to the effects of a single Lokomat session or conventional physiotherapy (Krewer et al. 2013). However, a case study involving two patients showed that GVS administered before five physiotherapy sessions led to an improvement in the SCP score (Nakamura et al. 2014). This finding is particularly interesting since no similar improvement was observed when the same five-session physiotherapy protocol (20 min of stimulation, anode on the ipsilesional side/cathode on the contralesional side, at a fluctuating intensity between 0.3 and 2.0 mA) was performed without prior GVS (Nakamura et al. 2014). Although the Barthel Index (BLS) improved in both configurations (with or without GVS), patients who underwent sessions with GVS seemed to perform better in sit-to-stand transitions, walking, and balance exercises. These observations, based on functional evaluations, were not consolidated with statistical analysis (Nakamura et al. 2014).

##### Postural balance

In the acute post-stroke phase, patients do not appear to be more sensitive to vestibular stimulation (binaural GVS of 35 s and 2 mA) than control subjects (Bonan et al. 2015). However, this is not the case for visual stimuli, which seem to be heavily relied upon by patients post-stroke to maintain balance, showing a highly significant difference compared to the control group. More than a month after the initial assessment, brain-injured patients are less unbalanced when faced with sensory disruptions (visual, proprioceptive, and vestibular). Therefore, the first few months following a stroke seem to be a period of individual perceptual-motor adaptation, with sensory recalibration likely playing a key role in this process (Bonan et al. 2015).

##### Subjective verticals

Left cathodal binaural GVS (fluctuating intensity between 0.4 and 1.5 mA), applied to patients with right hemisphere lesions, significantly improved all parameters of the subjective visual vertical assessment (Saj et al. 2006; Oppenländer et al. 2015b). Although improvements were observed in the parameters of the subjective tactile vertical, these were not statistically significant. These effects were primarily observed in subjects with mild deficits (Oppenländer et al. 2015b). No improvement in subjective vertical perceptions was noted in the placebo or right cathodal GVS groups (Oppenländer et al. 2015b). Moreover, the subjective visual vertical (SVV) could be modulated by reversing the polarity of GVS, regardless of whether the cathodal stimulus side was ipsilateral or contralateral to the lesion (Tomioka et al. 2022). In contrast, a study conducted by Volkening et al. (2018) on subjects with right-brain lesions found that GVS (left cathodal, right cathodal, and placebo, 10 to 12 daily stimulation sessions, 5 days/week, 20 min of stimulation at 1.5 mA) did not significantly improve the realignment of subjective visual and tactile verticals, either immediately or 2 and 4 weeks after stimulation. These findings appear to be contradicted by the study of Tomioka et al., which observed instantaneous modifications of the subjective visual vertical for different stimulation setups compared to placebo stimulation. However, no significant differences in outcomes were found between the two binaural stimulation patterns (Tomioka et al. 2022).

##### Spatial neglect

Spatial neglect is a clinical sign that may sometimes be present following a stroke localized in the right cerebral hemisphere. Recent studies have not focused on the effects of GVS on these sequelae, but earlier studies have addressed this issue. In patients with left hemispatial neglect following a right hemisphere stroke, 10–20 min of left cathodal GVS (fluctuating intensity between 0.4 and 2.0 mA for 20 min) significantly improved their scores on the line bisection test (Utz et al. 2011; Nakamura et al. 2015). In contrast, right cathodal GVS and the placebo condition showed no significant effects, whether measured 10 or 20 min after application (Utz et al. 2011; Nakamura et al. 2015). The effects of GVS on spatial neglect might be enhanced with higher stimulation intensities and longer application durations (Nakamura et al. 2015).

A preliminary study involving two patients with left hemispatial neglect suggested that right cathodal binaural GVS (fluctuating intensity of 1–1.5 mA for 20 min, over 5 days) could directly improve the target cancellation test, with effects lasting up to three days after stimulation (Zubko et al. 2013). However, no statistical analysis was performed, and the authors only observed the test results (Zubko et al. 2013). In contrast, two other studies, with the same stimulation parameters, showed no significant improvements in spatial neglect tests (form cancellation, reading, text copying, figure copying) in right-brain-injured patients after GVS (right cathodal, left cathodal, placebo) in the short and medium term (Ruet et al. 2014; Volkening et al. 2018).

Regarding egocentric and object-centered components, 20 min of left cathodal GVS (at 0.7 mA) significantly improved the line bisection and text copying tests in patients with left hemispatial neglect following a right stroke. Conversely, right cathodal GVS (at 0.7 mA) improved letter cancellation and figure copying tests. These effects were mainly observed in the most severely affected patients (Oppenländer et al. 2015a). No significant differences were found in non-neglecting or mildly neglecting subjects (Oppenländer et al. 2015a). Finally, the Barthel Index and the Behavioral Inattention Test showed highly significant improvements in patients with left hemispatial neglect, with effects persisting one month post-stimulation (Wilkinson et al. 2014). This stimulation was a binaural GVS with the cathode on the ipsilesional side (right mastoid), with a fluctuating intensity of 0.5–1.5 mA for 25 min, and was performed once a day for two weeks.

A recent systematic review (Wheeler et al. 2025) showed that the results of GVS on post-stroke spatial visual neglect were inconsistent and heterogeneous and required more rigorous methodological planning.

##### Sense of position and tactile extinction

In a 2013 study, Schmidt et al. (2013a) investigated the effect of GVS on position sense in left hemispatial neglect patients following right hemisphere strokes. When a left cathodal GVS was applied (intensity equal to the sensory detection threshold − 0.1 mA), patients showed significant improvements in position sense of their arm, lasting up to 20 min post-stimulation (Schmidt et al. 2013a). These results are striking, as the same stimulation applied to healthy subjects or right-brain-injured subjects without left hemispatial neglect did not result in any improvement in position sense (Schmidt et al. 2013a). Additionally, right cathodal GVS significantly worsened position sense in left hemispatial neglect patients (Schmidt et al. 2013a). The same research team also examined the effect of GVS on tactile extinction in right-brain-injured patients with left hemispatial extinction (Utz et al. 2011; Schmidt et al. 2013a). They found that 0.7 mA GVS significantly improved results on the Quality Extinction Test (QET), where patients were asked to recognize and discriminate between various stimuli (Schmidt et al. 2013b). These improvements were observed with both left and right cathodal GVS and appeared to persist over time, lasting up to 84 days post-stimulation (Utz et al. 2011; Schmidt et al. 2013b). The sham GVS applied to the control group had no significant effect on tactile extinction (Utz et al. 2011; Schmidt et al. 2013b).

#### Parkinson’s disease

According to recent reviews (Lee et al. 2021a; Wilkinson 2021), GVS is considered a low-cost, accessible, customizable, portable, and complementary approach to conventional Parkinson’s disease (PD) treatments.

##### Cognitively-motor task

Khoshnam et al. (2018) studied the progression of the finger tapping task (FTT) in patients with Parkinson's disease during the administration of binaural monopolar GVS (the cathodes on the mastoids and the anodes on the proximo-medial part of both forearms, at an intensity equal to twice the skin sensory threshold). The only statistically significant improvement observed in this study was in speed and rhythmicity (28.25%).

##### Static and dynamic postural instability

It appears that binaural GVS (cathode on the ipsilateral mastoid to trunk lateral flexion, with an intensity of 0.7 mA for 20 min) improved static postural instability in retroversion in some patients, with a more pronounced effect in those presenting significant postural deviation before stimulation (Kataoka et al. 2016). Regarding the anterior flexion angle in the standing position, binaural monopolar GVS (cathodes on the mastoid processes, and anodes on the trapezius muscles, with an intensity of 0.7 mA for 20 min) significantly improved this poor postural adjustment compared to placebo stimulation, whether the eyes were open or closed (Okada et al. 2015). For dynamic tests, the Timed Up and Go test showed a significant improvement of 16% when performed in association with GVS (Khoshnam et al. 2018). A recent meta-analysis studied the effects of GVS on postural disorders in Parkinson's patients, and it revealed that GVS has a favorable effect on postural balance in patients with Parkinson’s disease (Mahmud et al. 2022). However, due to the limited literature and inconsistent methodologies (variety of GVS intensities and waveforms), this favorable effect must be interpreted with caution (Mahmud et al. 2022).

##### Unified Parkinson's Disease Rating Scale (UPDRS)

Kataoka et al. (2016) showed in their study that binaural GVS (cathode on the mastoid on the same side of the trunk lateral flexion, with an intensity of 0.7 mA for 20 min) improved item 12 of the UPDRS (equivalent to the pull-test) in 3 out of 5 patients. For the other two patients, the score for this item remained unchanged (Kataoka et al. 2016).

As seen above, GVS is an efficient tool for counteracting PD symptoms, its use could be now extended to other symptoms of the disease, such as orthostatic hypotension, dyskinesia, and sleep disorders (Lee et al. 2021a).

#### Spinal cord injury

Very few studies currently exist on the potential benefits of binaural GVS in the treatment of spinal cord injuries. One suggests that this stimulation technique could, with 10 variable monophasic pulses from 1 to 10 mA and lasting 1 s each, reduce spasticity in some patients (5 out of 7) with an ASIA-A classification (Čobeljić et al. 2018).

#### Motion sickness

Motion sickness can be considered as normal physiological responses that can be elicited in almost all people, but susceptibility and severity can be high enough for the response to be considered a disorder in some cases. Therefore, motion sickness has been classified as a vestibular disorder by the Bárány Society (Cha et al. 2021). That being said, this functional disorder can be a handicap and a barrier in certain daily activities for some individuals (without structural issues).

Here, a first study on the use of GVS as a therapeutic tool for treating seasickness (Gutkovich et al. 2022) is reported. This pioneering study highlighted the beneficial effects of combining GVS with rotary chair treatment to reduce severe cases of seasickness. Three months after the intervention, participants in the treatment group showed a significant decrease in the number of visits to specialized clinics. They also reported a reduction in the consumption of motion sickness medications compared to the control group. Additionally, a significant reduction in the vestibular time constant (Tc) was observed, suggesting an improvement in the vestibular adaptation abilities of the treated patients. This study thus opens new perspectives for the use of GVS in managing seasickness, a field that has been little explored to date.

Moreover, GVS is also sometimes studied in combination with virtual reality and flight simulators to assess flight performance, secondary task performance, and simulator sickness (Pradhan et al. 2022). Twenty participants completed two separate VR flight simulation sessions, one with GVS and one without (control). The results would demonstrate the potential of synchronizing GVS with visual stimuli in virtual reality flight training to reduce visual-vestibular sensory conflict, thereby improving fidelity and performance.

#### Peripheral vestibular disorders

In addition to central diseases as seen above, some studies have also focused on peripheral vestibular disorders, with a growing interest in the use of GVS as a rehabilitation tool for these conditions.

These studies examined the effects of GVS on various outcomes related to balance and vertigo in patients with peripheral vestibular disorders (unilateral or bilateral). The study by Ceylan et al. (2021) on unilateral peripheral vestibular disorders showed better balance scores (sensory organization test) and lower levels of vestibular discomfort, as assessed by the Visual Analogic Scale (VAS). Presumably, the same results are observed in patients with bilateral vestibulopathy, with improvements in the VAS (for vertigo and walking assessment), the Activities-specific Balance Confidence Scale (ABC), and the Scale for Assessment and Rating of Ataxia (SARA) (Nguyen et al. 2022).

Overall, these studies highlight the positive effects of GVS on balance, walking, and the perception of vestibular movements in various patient groups. However, the results also suggest that the effectiveness of GVS may vary depending on several factors such as the patient's condition at the time of treatment, whether it is combined with vestibular rehabilitation or or the type of stimulation used.

#### Noisy GVS: a promising tool for rehabilitation?

Noisy GVS has emerged over the past decade as a promising technique for clinical use. It has been widely used in studies involving healthy participants, particularly for its effects on postural control and balance (McLaren et al. 2023; Xie et al. 2024). The observed improvement in balance performance in these populations has sparked increasing interest in its therapeutic potential. This growing attention is reflected in the rising number of clinical studies exploring nGVS in various pathological conditions. The principal findings from these studies have been synthesized in several recent systematic reviews (e.g. Pires et al. 2022; McLaren et al. 2022), highlighting the potential clinical benefits of nGVS in diverse patient populations.

Noisy GVS has been used in numerous clinical trials for the functional treatment of Parkinson's disease. In a manual tracking task, Lee et al. (2015) demonstrated that the error between the target position and the cursor position significantly decreased when nGVS was applied (statistical comparison between GVS-ON and GVS-OFF). More recently, the same team conducted a study on the frequency-specific effects of GVS on response-time performance in the same population of Parkinson's patients (Lee et al. 2021a, b). Using band-limited GVS (ms-GVS), they found that the stimulation frequency yielding the best response time varied considerably across participants and exceeded 30 Hz in half of the Parkinson's patients in the "off" phase without levodopa medication. Moreover, a simple case report indicates that binaural nGVS might lead to an increase in functional independence, motor and sensory levels, as well as improvements in postural balance and trunk control in this type of neurological injury (Nascimento and Boffino 2022). Finally, binaural nGVS in this same patient population would lead to a beneficial response in terms of balance. However, nGVS had no effect on normal gait parameters compared to placebo stimulation (Peto et al. 2024).

Weech and his collaborators examined the impact of nGVS on reducing cybersickness during virtual reality exposure (Weech et al. 2020). Participants played two different virtual reality games, classified as moderately or intensely nauseogenic. The exposure to noisy GVS (± 1750 μA) or sham stimulation (0 μA) lasted for 30 min, with 10 min of gameplay before and after this exposure. The results suggest that noisy GVS could potentially help reduce cybersickness under intense VR conditions.

Wuehr et al. (2023) found that GVS was particularly effective in improving impaired vestibular motion perception in patients with bilateral vestibulopathy, especially in those with poor baseline perceptual performance. In a follow-up study (Wuehr et al. 2024), they observed that the reductions in body sway induced by GVS were consistent with stochastic resonance, further supporting its effectiveness in improving balance. However, Eder et al. (2022) found no significant difference between the groups (nGVS + vestibular rehabilitation therapy versus sham + vestibular rehabilitation therapy-VRT), suggesting that GVS provided no additional benefit when combined with VRT treatment (Chen et al. 2021).

## Discussion

The present review emphasized the popularity of GVS and the diversity and richness of the studies using or related to this investigation tool. It can be observed that most of these studies, including the latest, deal with GVS in itself as well as the topic tackled using it. Many reviews have already been published on the subject, recently focused on either clinical use of GVS (e.g. Pires et al. 2022; McLaren et al. 2022), or its parameters and configuration (Valter et al. 2025). In this context, the present narrative review spans from the historical foundations and fundamental principles of GVS to its applications in both basic and clinical sciences, thus providing an updated overview that complements and extends previous reviews using a similar angle of view (e.g. Fitzpatrick and Day 2004; St George and Fitzpatrick 2011; Dlugaiczyk et al. 2019). This discussion will first cover what is now well established concerning GVS and then open to concepts that deserve further investigation.

Animal studies have clearly shown that GVS acts at the level of the vestibular nerve fibers, coming from all components of the vestibular apparatus—including both the semicircular canals and the otolithic organs (Forbes et al. 2023). This distinguishes GVS from other vestibular stimulation techniques such as caloric vestibular stimulation, which only activates the semicircular canals, particularly the lateral canal, or sound-induced vestibular stimulation, which mainly targets the utricle and the saccule.

Most of GVS parameters are finely adjustable. To begin with, it is worth taking advantage of the fact that akin natural vestibular stimulation which can either enhance or decrease afferents discharge rates, both cathodal and anodal currents act on vestibular fibers: the former increasing, the latter decreasing discharge frequency at onset, cutting off the current inducing an opposite frequency of discharge modulation. Interestingly, in both cases asymmetrical modulation occurs: activation being larger than inhibition (Forbes et al. 2023). All this deserves thorough consideration in future GVS protocols.

Considering cathodal and anodal opposite effects, it can be claimed that a crucial parameter is how polarized both vestibular apparatus are. Postural studies using binaural bipolar or alternative configurations confirm this hypothesis (Séverac Cauquil et al. 2000; Aoyama et al. 2015). Further oculomotor studies using other montages than the classic binaural bipolar could help in demonstrating the effect of polarization on the direction of GVS-induced effets.

Ocular studies allow us to interpret the influence of stimulation intensity. Indeed, the amplitude of eye deviations, along the 3 axes of rotation, increase with stimulus intensity to a certain extent until a threshold is reached from where the response either reaches a plateau or triggers nystagmus. This should lead GVS users, at least in healthy volunteers, to go for low intensities. In that case, the responses are pure and not constrained by biomechanical limits, and moreover, tactile sensation is either absent or very mild. This condition should constitute a requisite in terms of ethics, subjects respect and data reliability, ensuring the absolute blindness of the subject regarding the occurrence and the direction of the stimulation.

Studies combining GVS and fMRI have significantly contributed to identifying cortical networks involved in the perception of self motion with a specific direction, particularly through the use of directional GVS protocols (Smith et al. 2012; Aedo-Jury et al. 2020). The networks largely overlap with those activated by visual motion stimulation (Morrone et al. 2000; Cardin and Smith 2010; Pitzalis et al. 2013, 2015), such as optic flows, suggesting a shared neural basis for vestibular and visual motion integration. Directional GVS therefore emerges as a relevant tool to probe the neural substrates of perceived direction, heading, and locomotion in neuroimaging paradigms. However, the ability to induce a realistic self motion perception experience may be limited by the supine position required in the fMRI, which does not reflect natural conditions for experiencing self-motion. To address these limitations, fNIRS has emerged as an interesting alternative, allowing data acquisition in more naturalistic postures, in spite of only measuring superficial cortical structures (Hernández-Román et al 2023). Electroencephalography (EEG) and magnetoencephalography (MEG) could also be considered, given its high temporal resolution, to further investigate the dynamics of visuo-vestibular integration in self motion perception (Burgess 2020). However, stimulation-induced electrical artifacts can challenge the data collection and analysis.

The perception of self-motion induced by GVS is ultimately the subjective correlate of the cortical activations measured in neuroimaging studies. However, capturing and evaluating this percept remains a significant challenge. The limited number of studies underlines the methodological difficulties in objectively measuring the percept induced by GVS, which may partly account for the observed interindividual variability (Gallagher et al. 2023; Houben et al. 2024). This issue is further complicated by the fact that participants often struggle to consciously identify and articulate the sensation induced by GVS.

The percept is influenced by several stimulation parameters, including waveform, duration, and intensity. Unlike postural and oculomotor responses, which occur rapidly and reflexively, the perceptual experience of motion emerged over a longer timescale (Previc and Mullen 1990). These temporal dynamics must be considered when designing GVS protocols aimed at eliciting a clear subjective experience. In this context, individualized stimulation appears essential. As emphasized by Valter et al. (2025) in their recent review on GVS parameters, adjusting stimulation intensity to individual thresholds is generally recommended to optimize the effects of GVS across different modalities. As Gallagher et al. (2023) suggested, this approach may also improve the reliability of perceptual responses by ensuring that stimulation exceeds the necessary threshold for each participant. Applying such a personalized threshold to studies on posture or eye movements could be particularly valuable, as these domains also show substantial interindividual variability in response amplitudes (MacDougall et al. 2002).

Furthermore, such an approach could help address unresolved questions regarding the distribution and functional organization of vestibular pathways. It remains unclear whether the electrical signal delivered by GVS is equally distributed across these pathways, to what extent they operate independently, and whether certain pathways may be more dominant in some individuals.

Despite encouraging findings, clinical studies investigating GVS in central and peripheral neurological disorders remain highly heterogeneous. Differences in protocols, disease stages, and lesion severity make direct comparisons more challenging, and the sample sizes, which are often limited to case reports, drastically preclude generalizability. Most of the studies differ with respect to methodology and are uncontrolled and nonrandomized. This reflects the fact that GVS remains an underexplored area in clinical research. Additionally, the long-term effects of GVS remain poorly understood, as most studies investigate only the immediate responses to one session of stimulation.

In clinical studies, nGVS has recently gained interest for its potential therapeutic applications, especially in balance and postural control. While some studies support its use in neurological populations like Parkinson's and bilateral vestibulopathy, the findings remain inconclusive. The inconsistency in stimulation parameters and individual responses has prevented the establishment of a standardized application protocol, and most studies do not report effects greater than what can be achieved through existing rehabilitation techniques.

## Conclusion

Further research needs to be directed towards optimizing protocols and standardizing stimulation parameters, which could involve adjusting the stimulus to each individual, in order to strengthen the GVS paradigm. Now, while postural responses to directional GVS have been extensively documented, other aspects are not fully resolved yet, such as oculomotricity. Considering neuroimaging and perceptual effects, the variety of current studies underlines the richness of possibilities yet to be explored and questions yet to be addressed. Directional GVS remains largely unexplored in the therapeutic field, and could, in the future, benefit from the contributions of basic science findings. Major progress has undoubtedly been made, but a coordinated effort between basic research and clinics to lead well-controlled large-scale studies is crucial to make the most of the full therapeutic potential of GVS.

## Data Availability

No datasets were generated or analysed during the current study.
